# Deployment and uptake of COVID-19 vaccines for refugees and migrants in regular and irregular situations: a mixed-method multicountry study

**DOI:** 10.1136/bmjopen-2024-087629

**Published:** 2025-02-02

**Authors:** Pierina Benavente, Vinay N Kampalath, Moussa Lonkila Zan, Nguyen Toan Tran, Elżbieta Anna Czapka, Seyed-Moeen Hosseinalipour, Enrique Teran, Cheryl Martens, Biraj Man Karmacharya, Anjali Joshi, Jai K Das, Zahra A Padhani, Vicente B Jurlano, Maria Midea M Kabamalan, Laetitia Nyirazinyoye, Karl Blanchet

**Affiliations:** 1 Department of Global Public Health and Primary Care, Faculty of Medicine, Pandemic Centre, Bergen, Norway; 2 Department of Paediatrics, Children’s Hospital of Philadelphia, Division of Emergency Medicine, Philadelphia, Pennsylvania, USA; 3 University of Pennsylvania Perelman School of Medicine, Philadelphia, Pennsylvania, USA; 4 Institut Supérieur des Sciences de la Population, Ouagadougou, Centre, Burkina Faso; 5 University of Geneva Faculty of Medicine, Geneve, Switzerland; 6 Australian Center for Public and Population Health Research, University of Technology Sydney, Sydney, New South Wales, Australia; 7 Sociology Institute, University of Gdańsk Faculty of Social Sciences, Gdansk, Pomorskie, Poland; 8 Geneva Centre of Humanitarian Studies, Faculty of Medicine, University of Geneva, Geneva, Switzerland; 9 Geneva Cenre of Humanitarian Studies, Faculty of Medicine, University of Geneva, Geneve, Switzerland; 10 Universidad San Francisco de Quito, Quito, Ecuador; 11 Instituto de Estudios Avanzados en Desigualdades, Universidad San Francisco de Quito, Quito, Pichincha, Ecuador; 12 Kathmandu University, Dhulikhel, Nepal; 13 Department of Public Health, Kathmandu University School of Medical Sciences, Kathmandu, Nepal; 14 Division of Women and Child Health, Aga Khan University, Karachi, Pakistan; 15 Institute for Global Health and Development, Aga Khan University, Karachi, Pakistan; 16 School of Public Health, University of Adelaide, Adelaide, South Australia, Australia; 17 The Demographic Research and Development Foundation, Inc, Quezon City, Philippines; 18 College of Social Sciences and Philosophy, University of the Philippines Diliman, Quezon City, Metro Manila, Philippines; 19 School of Public Health, Department of Community Health, University of Rwanda College of Medicine and Health Sciences, Kigali, Rwanda; 20 Geneva Centre of Humanitarian Studies, Faculty of Medicine, University of Geneva, Geneva, GE, Switzerland

**Keywords:** COVID-19, Vaccination, Health policy, Refugees, Health Equity, Vulnerable Populations

## Abstract

**Abstract:**

**Background:**

The COVID-19 pandemic has widened inequities, affecting migrant and refugee populations in vulnerable situations, who may face elevated risks of infection, constrained healthcare access and discrimination. Inclusive vaccination campaigns are recommended, but barriers persist. This study aimed to identify barriers and facilitators and estimate vaccination coverage among refugees and migrants in low- and middle-income countries, emphasising inclusive policies for effective rollout.

**Methods:**

A mixed-method study was conducted in two phases in Ecuador, Nepal, Pakistan, the Philippines and Rwanda. Phase 1 (March–May 2022) included policy analysis, in-depth interviews and focus-group discussions with 52 key informants analysed with thematic and grounded theory approaches using hybrid coding. Phase 2 (June–August 2022) included a cross-sectional study among refugees and migrants in regular (MIRS) and irregular situations (MIIS) and used descriptive analysis and a COVID-19 Vaccine Equity Index (CVEI).

**Results:**

A total of 1378 individuals responded to the survey (43.8% MIRS, 31.2% MIIS and 25% refugees). 87% reported receiving at least one dose of the COVID-19 vaccine. The CVEI at the global level (0.824) suggested differences in complete vaccination between migrants and other residents in most of the study countries (refugees reported more access to vaccines than MIRS and MIIS). However, the qualitative phase reported delays and inequities in the early stage of the vaccination process in all countries. Overall, 64.2% of respondents perceived that government’ campaigns were successful. Both the qualitative and quantitative phases identified several barriers and facilitators. The main barriers included a lack of trust in authorities, extended waiting times and distance to vaccination centres, discrimination and xenophobia, lack of identity documentation, and adverse reaction concerns. On the other hand, the primary facilitators were the widespread distribution of vaccination centres, sources and provision of information, specific campaigns for refugees and migrants, free vaccination and the motivation to protect others’ health.

**Conclusions:**

Despite the high coverage of COVID-19 vaccines among refugees and migrants in the study countries, the process had significant barriers. Simple vaccination registration procedures, targeted campaigns, mobile vaccination teams for hard-to-reach and vulnerable groups, and building trust in the host country authorities are pivotal for future and inclusive vaccine deployments.

**STRENGTHS AND LIMITATIONS OF THIS STUDY**

- The comprehensive data collection approach successfully included a broad range of migrant populations, even those that are typically hard-to-reach migrants in irregular situations (MIIS) group.
- Our study is the first multicountry mixed-method study specifically evaluating COVID-19 vaccination coverage among refugees and migrants.
- The study used various recruitment methods to reach hard-to-access groups, including snowball sampling, which may have resulted in a non-representative MIIS sample.
- Reliance on self-reported information, including vaccination status, possesses risks of recall bias or information concealment, impacting data accuracy.
- The absence of stratified analysis by key characteristics may mask important variations within the sample.

## Background

 The COVID-19 pandemic impacted the global population and widened inequities between refugees and migrants and the general population.^1^ In countries with available data, primarily high-income countries, migrants were at higher risk of being infected by SARS-CoV-2 and were disproportionately represented in hospitalisations and fatalities.^2^ The situation is even more salient when considering migrants in irregular situations (MIIS) whose access to healthcare is often constrained by fear of being identified and deported, inability to speak the local language, lack of knowledge to navigate the local health system, and social isolation.^3^

Including migrants in vaccination campaigns has been recommended as a public health priority, together with the need for more research.^4^ Past research on immunisation coverage demonstrates structural-level barriers to vaccine uptake among refugees and migrants (box 1), including disruption of health service delivery in the context of large-scale migration, as well as individual-level barriers to access, such as knowledge, attitudes and practices.^5^ Together with challenges due to their legal status, migrants may also experience interpersonal and institutionalised discrimination, which can further compromise vaccine accessibility.^6^ Importantly, they may face obstacles to health and vaccination systems in the new host country due to a lack of entitlements, administrative barriers and linguistic challenges.

Box 1 **Key definitions**

Refugee—refugees are persons who are outside their country of origin for reasons of feared persecution, conflict, generalised violence or other circumstances that have seriously disturbed public order and, as a result, require international protection.^43^

Migrants—a term used in this paper to designate both MIRS and MIIS.

Migrants in regular situations—individuals whose migration occurs in compliance with the laws of the country of origin, transit and destination.^44^

Migrants in irregular situations—individuals whose movement takes place outside the laws, regulations or international agreements governing the entry into or exit from the state of origin, transit or destination.^44^

Recent studies highlight the importance of tailored vaccination models to meet the needs of diverse populations, emphasising the need for flexible funding, outreach, translation and culturally responsive services to address ongoing and emergent factors such as vaccine hesitancy.^7^ Considering the slower and lower levels of uptake among migrants in irregular situations, even in the few European countries who provided equal access to COVID-19 vaccines, shows that sole focus on policy commitments cannot guarantee uptake and adoptive vaccine programmes for different subcommunities are needed.^8^ Some recent findings indicate delayed, overdue or missed follow-up COVID-19 shots among non-European migrants and resettled refugees in England, especially among older and non-white groups, indicating a need for further research.^9^ Moreover, lower perception of infection and lower integration level into host country are also among reasons for lower vaccine acceptance.^10^ Additionally, the WHO has published interim guidance on COVID-19 immunisation in refugees and migrants, outlining key principles and policy considerations for equitable prioritisation and access to vaccines.^11^ These efforts underscore the importance of community engagement, communication to build trust, and innovative approaches for vaccine delivery.^11^

Studies on refugees and migrants and COVID-19 vaccines have also focused on the high level of vaccination hesitancy among different migrant groups.^12^,^14^ In addition, there is evidence of the various obstacles faced by COVID-19 vaccination campaigns:^15^ (1) difficulty in reaching migrant populations; (2) lack of adaptation in the format and language of national vaccination campaigns; and (3) lack of knowledge of migrants of their rights in the host country.^16^ The gap of evidence about COVID-19 vaccine coverage and the social and political determinants of COVID-19 vaccination among refugees, migrants in regular situations (MIRS), and MIIS precludes advocacy towards inclusive vaccination policies and undermines effective rollout.

This study was conducted in Ecuador, Nepal, Pakistan, The Philippines and Rwanda, five low- and lower-middle-income countries (LLMICs) impacted by large-scale migration. We aimed to investigate the barriers and facilitators to COVID-19 vaccination among refugees, MIRS or MIIS and to estimate COVID-19 vaccination coverage among these three different migrant groups. Additionally, we also aimed to estimate the proportion of refugees and migrants reached by COVID-19 vaccination campaigns and measure the magnitude of inequity compared with the general population.

## Methodology

A phased mixed-method study was conducted in two consecutive phases: (1) phase 1 (policy analysis and qualitative study) performed between March and May 2022 and (2) phase 2 (a cross-sectional study) between June and August 2022 (online supplemental figure 1 in Appendix page 1 shows the overall study methodology).

The overall study is grounded in the WHO Health Systems Framework with its six-system building blocks and the Behavioural and Social Drivers (BeSD) of COVID-19 Vaccination Framework.^17 18^ The six-system building blocks are leadership and governance, healthcare financing, health workforce, medical products and technologies, information and research, and service delivery. This framework informed the design and contents of our surveys and helped enhance the actionability of our findings in terms of health system strengthening. The BeSD of COVID-19 Vaccination Framework allows careful consideration of how individual, social and structural determinants interact to impact willingness to vaccinate and vaccination uptake (online supplemental figures 2 and 3 in Appendix pages 1 and 2 show the study frameworks).

Patients and the public were not involved in the design, conduct or reporting of the study.

### Study countries

The five study countries are LLMICs known to host a significant number of refugee and/or migrant populations. They comprise five of the six WHO regions and had a relatively high COVID-19 vaccination coverage among the general population (a vaccination coverage above 50% of the total general population). A snapshot of each country’s general, refugee and migrant populations and COVID-19 vaccine coverage as of 30 March 2022 is detailed in the online supplemental table 1 (Appendix page 2).

The University of Geneva worked in each country with public health research institutes that could deploy experienced researchers and had access to key informants. All country research partners contributed to developing the methodology and the data collection tools, which were common to all countries. Online supplemental table 2 in the Appendix (Appendix page 2) details each country’s sites and lead researchers.

### Phase 1: policy analysis and qualitative study

#### Study designs

In phase 1, a qualitative approach was adopted, and in-depth interviews (IDIs) and focus-group discussions (FGDs) with key informants (KIs) were conducted. For the data and policy analysis, we gathered all global and national data about COVID-19 vaccinations and other variables related to refugees and migrants. In addition, an analysis of national vaccination policies in each country was performed to complement and triangulate the findings from the IDIs and FGDs (online supplemental figure 1 in Appendix page 1).

At the country level, the study engaged a team of social scientists with relevant experience in qualitative methods. The interviews were conducted in local languages, in person, remotely via phone, or videoconference, depending on the pandemic situation in each country and the safety protocols at partner institutions. For all IDIs and FGDs, privacy was guaranteed by national researchers. Semistructured interview guides were designed based on different guidelines and validated resources.^18^,^24^ The guides were used by all countries for IDIs and FGDs. They included questions about barriers to and facilitators of COVID-19 vaccination among refugees, MIRS and MIIS. The interviews were audiotaped after obtaining consent from participants.

#### Participants and sampling

For phase 1, KI and FGD participants were 18 years old and above and were not excluded based on gender, race, religion or ethnicity. The participants were community leaders or stakeholders from NGOs and governments involved in COVID-19 vaccine implementation and/or able to provide relevant perspectives and insights on COVID-19 vaccine access and uptake among refugees and migrants. All participants provided consent. 2 FGDs and 42 IDIs were conducted. Table 1 summarises the number and type of participants by country.

**Table 1:** Number and description of study participants in phase 1 by country, city and employer

Participant	Total
Ecuador—key informant interviews	
Local NGOs working with migrants	3
Health-related international agency	1
Vaccination programme at Ministry of Health	1
University vaccine programmes	4
Health authority of City of Quito	1
Total, Ecuador	10
Nepal—key informant interviews	
Municipal Health Office	3
International agency	4
Department of Health	1
Total, Nepal	8
Philippines—key informant interviews	
Department of Health	1
Migrant association	1
Department of Foreign Affairs	2
United Nations agency	2
Research centre on migration	1
International agency	1
Commission on Population and Development	1
Municipal Health Office	1
Total, Philippines	10
Pakistan—key informant interviews	
Local NGO working with migrants	1
Vaccination office	1
Local COVID-19 response organisation	1
Health department	1
Health department	1
Community leader	2
United Nations agency	1
Pakistan—focus-group discussions	
Community leaders	10
Total, Pakistan	18
Rwanda—key informant interviews	
United Nations agency	3
Ministry of Health	2
Local NGO working on health	1
Total, Rwanda	6
Grand total	52

A non-probabilistic expert sampling strategy was adopted. This type of purposive sampling approach is helpful in the early phases of a research project to gain in-depth insights into an issue, explore key themes and inform further avenues of inquiry.^25^ In-country research partners and collaborating local organisations helped establish a sampling frame of key informants based on their knowledge and professional network.

#### Data analysis

For the IDIs and FGDs, research assistants transcribed and translated the audio records into English. Accuracy checks were done by comparing transcripts with audio files.

The analysis was based on a thematic and grounded theory analysis using hybrid coding. This approach used a codebook, initially constructed based on published literature (deductive method), and then enriched with new emerging nodes from the study data (inductive method) during the manual coding process. Consensus coding was used to enhance the trustworthiness of the analysis. The researchers from the national teams and the core research group coded the same transcripts and compared results.

The policy analysis covered government documents, policy statements, vaccination programmes and other relevant documents. All policies formulated during the pandemic available on governmental websites and other institutional websites such as the UNICEF and the United Nations High Commissioner for Refugees (UNHCR) for curtailing the spread of COVID-19 and for vaccination rollout were reviewed and summarised. The policy documents were analysed for their inclusiveness and exclusiveness concerning MIRS, MIIS and refugees. Instead of conducting a formal content analysis, specific aspects of the policies were reviewed and compared among countries.

### Phase 2: a cross-sectional study

#### Study design

A descriptive cross-sectional study was conducted in every country using a questionnaire comprising five different sections: sociodemographic characteristics, health status related to COVID-19, and preventive measures, vaccination coverage, BeSDs (including barriers), and vaccination campaigns (online supplemental figure 1 in Appendix page 1). The survey was designed based on guidelines and validated resources.^18^,^24^ The same tool was used across all the countries, starting with a pilot test in Ecuador. Each national research institute added a few questions to refine their analysis based on their national context.

#### Participants and sampling

For phase 2, participants were adults 18 years of age and above, self-reported belonging to the refugee, MIRS or MIIS communities, and agreed to participate in the study by providing informed consent. No participant was excluded based on gender, race, religion or ethnicity.

Different outreach strategies were employed according to the distribution of the three study populations across the different countries. Official registries constituted the starting point for identifying and recruiting refugees and MIRS. Refugees are commonly registered in official databases managed by the national government and relevant United Nations agencies, such as UNHCR. MIRS may also be found in official government databases or IOM records. In contrast, identifying and recruiting MIIS, who are often not included in official registries, required a different approach. Snowball sampling was used for the recruitment of MIIS.

To enhance the rigor of our quantitative sampling and reduce bias, we implemented a range of strategies tailored to the specific circumstances and challenges present in each country. For example, in some countries, convenient sampling was employed due to accessibility and proximity, while in others, initial seeds provided by humanitarian organisations facilitated snowball sampling. Additionally, purposive sampling was used in cities with high concentrations of migrant populations.

The refugee population for our sample size calculation was used as data on refugees are more readily available than data on MIRS and MIIS. Our estimation was based on the following explicit assumptions: (1) the proportion of fully vaccinated refugees was assumed to be a quarter of that of the general population, drawing from diphtheria, tetanus and polio (DTP) vaccine coverage studies in Jordan and Lebanon,^26 27^ and this assumption was applied to all six countries, no matter their current COVID-19 vaccine coverage and age distribution; and (2) the full-vaccination coverage in the general population was used as a proxy for full-vaccination coverage across all adult age strata within that population.

#### Outcomes measures and variables

The primary outcomes were the proportion of refugees vaccinated against COVID-19, the proportion of MIRS vaccinated against COVID-19, the proportion of MIIS vaccinated against COVID-19, and the COVID-19 Vaccine Equity Index (CVEI). The CVEI was developed based on existing indices, such as the Vaccine Equity Index by Maul *et al*^28^ and the CVEI by Pressman *et al*.^29^ The CVEI measures the proportion of unvaccinated individuals in each subgroup relative to the total population.

For all subgroups,

$$\text{CVEI}=\frac{P\left(U_{s}\right)}{P\left(U_{t}\right)}$$

where *U*=number of unvaccinated, *s*=subgroup and *t*=total population. If CVEI>1 for a given subgroup, that group receives unequal and less favourable treatment, whereas a CVEI<1 implies unequal but favourable treatment. A CVEI of 0 means the subgroup has 0% of unvaccinated (or is entirely vaccinated). To calculate CVEI, the proportion of vaccinated in each country was used, as shown in online supplemental table 1 of the Appendix (page 3). For the global CVEI, a mean of the proportions of single countries weighted by the countries’ population sizes was calculated. The calculation of the CVEI is based on several key assumptions. First, we assumed the vaccination data in each country are accurate and complete. Second, we assumed that using population sizes to weigh the proportions for calculating the global CVEI accurately reflects the true global distribution. Finally, we assumed all subgroups are similar for this calculation, even though we recognise there could be differences among them.

#### Ascertainment of vaccination status

The gold standard of seeing proof of vaccination may not be feasible for different reasons. For instance, participants may not have proof readily available at the time of the interview, or the interview may be done via phone. Asking for such proof from our study population could be interpreted as intrusive and may deter participants from completing the survey. Therefore, the vaccination status was based on participants’ oral self-reporting. The survey noted whether data collectors verified visual proof of vaccination.

#### Data collection and analysis

All countries except the Philippines used KoboToolbox to collect and upload survey data. Data were collected using the Census and Survey Processing System (CSPro4) in the Philippines. Country teams did the country-level analyses with the support of the University of Geneva team, which was also responsible for the global analysis and report. Missing values based on immigration status were dropped. These analyses followed an analysis plan using descriptive analysis (simple distribution and cross tables), the CVEI and χ^2^ tests to compare subgroups. For the descriptive analysis, simple frequencies and percentages by country were computed for all critical variables, but when relevant, variables were cross-tabulated with gender and migration status. We also performed analysis to compare countries and categories using confidence intervals.

## Results

### Sociodemographic and risk of COVID-19 infection

In total, 1378 individuals were surveyed across the five different countries, but this sample was not equally distributed by country: Pakistan (499), Ecuador (334), Nepal (210), Rwanda (205) and the Philippines (120) (table 2). Men and women were equally represented in the final sample. Only Nepal and the Philippines had less than 50% of women among the respondents (accounting for 42.9% and 20.8% of the countries’ respondents, respectively). On the other hand, female respondents are more represented in Ecuador, Pakistan and Rwanda. Globally, 43.8% of respondents were MIRS. Migration status differed between countries, reflecting the unique nature of migration in each country. For example, 68.9% of respondents were MIIS in Ecuador, 68.9% were MIRS in Pakistan and 75.1% were refugees in Rwanda. The proportion of MIIS was very low in Rwanda (2%), and the proportion of refugees was 4.9% in Ecuador.

**Table 2:** Sample distribution by country and gender, migration status, age, duration in the country, and education

	Overall sample	Ecuador	Nepal	Pakistan	Philippines	Rwanda
	N (%)	n (%)	n (%)	n (%)	n (%)	n (%)
Gender						
Female	684 (49.6)	177 (51.4)	90 (42.9)	259 (51.9)	25 (20.8)	133 (64.9)
Male	691 (50.1)	164 (47.7)	120 (57.1)	240 (48.1)	95 (79.2)	72 (35.1)
Other	3 (0.2)	3 (0.9)	0 (0.0)	0 (0.0)	0 (0.0)	0 (0.0)
Migration status						
Regular	603 (43.8)	90 (26.2)	66 (31.4)	344 (68.9)	56 (46.7)	47 (22.9)
Irregular	430 (31.2)	237 (68.9)	43 (20.5)	113 (22.6)	33 (27.5)	4 (2.0)
Refugee	345 (25.0)	17 (4.9)	101 (48.1)	42 (8.4)	31 (25.8)	154 (75.1)
Age group						
18–29	354 (24.4)	114 (33.2)	46 (21.9)	84 (14.7)	85 (70.8)	25 (12.2)
30–49	503 (34.7)	169 (49.1)	65 (30.9)	116 (20.4)	29 (24.2)	124 (60.5)
50–64	288 (19.9)	52 (15.1)	52 (24.8)	141 (24.7)	5 (4.2)	38 (18.5)
65+	233 (16.1)	9 (2.6)	47 (22.4)	158 (27.7)	1 (0.8)	18 (8.8)
Duration in the host country						
< 1 year	117 (8.4)	86 (25.0)	11 (5.2)	17 (3.4)	2 (1.7)	1 (0.5)
1–5 years	328 (22.7)	208 (60.5)	14 (6.7)	7 (1.4)	70 (58.3)	28 (13.7)
More than 5 years	933 (68.9)	50 (14.5)	185 (88.1)	475 (95.2)	48 (40.0)	175 (85.8)
Education						
Formal education not completed	426 (30.9)	7 (2.0)	25 (11.9)	360 (72.1)	0 (0.0)	34 (16.5)
Primary education	152 (11.0)	23 (6.7)	42 (20.0)	58 (11.6)	0 (0.0)	29 (14.1)
Secondary education completed	235 (17.1)	127 (36.9)	29 (13.8)	29 (5.8)	35 (29.2)	16 (7.8)
Secondary education not completed	118 (8.6)	41 (11.9)	30 (14.3)	19 (3.8)	2 (1.7)	26 (12.6)
Technical education	23 (1.7)	18 (5.2)	0 (0.0)	0 (0.0)	1 (0.8)	4 (1.9)
Bachelor’s degree	197 (14.3)	93 (27.0)	8 (3.8)	5 (1.0)	68 (56.7)	23 (11.2)
Postgraduate degree	35 (2.5)	8 (2.3)	1 (0.5)	0 (0.0)	14 (11.7)	12 (5.8)
None of the above	154 (11.2)	12 (3.5)	75 (35.7)	17 (3.4)	0 (0.0)	50 (24.3)
I prefer not to answer	38 (2.8)	15 (4.4)	0 (0.0)	11 (2.2)	0 (0.0)	12 (5.8)
Total	1378 (100)	344 (100)	210 (100)	499 (100)	120 (100)	205 (100)

Among all respondents, 67.7% (95% CI 65.2% to 70.1%) stayed in their host country for more than 5 years, while in Ecuador, only 14.5% (50/344) lived there for more than 5 years. 31% of respondents (426/1378) did not complete a formal education (without any primary education). The Pakistani sample mainly influences this proportion, in which 72.1% (360/499) did not complete formal education. Globally, 70.1% (966/1378) of respondents did not report high-risk conditions (age 60+ years, diabetes, heart disease or high blood pressure, chronic lung diseases, weak immune system and obesity). Few high-risk groups were represented in the country samples (online supplemental graph 1 in Appendix page 3).

Overall, 15.4% (212/1378) of the respondents reported having had COVID-19 infection, including 14.0% (96/684) of women and 16.5% (114/691) of men. The infection rates were the highest in Ecuador, regardless of gender and migration status, with a third of the people surveyed reported to have been infected once (33.4% (115/344)). Men in Ecuador were more likely to be infected compared with women (37.8% (62/164) vs 28.8% (51/177)) (online supplemental table 3 in Appendix page 3).

### Vaccination coverage

Overall, 86.6% ((1194/1378), 95% CI 84.7% to 88.3%) of the respondents were vaccinated with at least one dose; the highest vaccination coverage was in Rwanda (99% (95% CI 96.2% to 99.8%)) and the lowest in Pakistan with 69.7% (95% CI 65.5% to 73.6%). Pakistan accounted for 82% (151/184) of the total unvaccinated participants. Overall, fewer women (83.8% (573/684), 95% CI 80.8% to 86.4%) reported being vaccinated than men (89.4% (618/691), 95% CI 86.9% to 91.5%, p<0.001). MIRS had a lower vaccination rate (81.3% (490/603)) in comparison to MIIS (88.6% (381/430), 95% CI 85.2% to 91.3%, p<0.001) and refugees (93.6% (323/345), 95% CI 90.5% to 95.8%, p<0.001). Respondents without formal education were less likely to be vaccinated (Pearson χ^2^(8) = 111.5286, p<0.001). In Ecuador, Pakistan and the Philippines, refugees tended to have less access to COVID-19 vaccines than MIIS and MIRS (see online supplemental table 4 in Appendix page 4). Following the IDIs and FGDs, the qualitative analysis revealed that most KIs were uncertain about estimating COVID-19 vaccination coverage among migrants and refugees in their respective countries.

Among the 1378 respondents, 67.5% ((462/684), 95% CI 63.9% to 71.0%) of women received full doses of COVID-19 vaccine, while this rate is 64.2% ((444/691), 95% CI 60.6% to 67.7%) among men. The rate of vaccine series completion was 79.7% ((275/345), 95% CI 75.1% to 83.6%) among refugees, 61.6% ((265/430), 95% CI 56.9% to 66.1%) among MIIS and 60.9% ((367/603), 95% CI 56.9% to 64.7%) among MIRS. The Philippines (96.7% (116/120), 95% CI 91.4% to 98.7%) and Rwanda (96.1% (197/205), 95% CI 92.4% to 98.0%) had the highest rates of full vaccination compared with the other countries (p<0.001), while only 47.3% ((236/499), 95% CI 42.9% to 51.7%) of individuals in Pakistan received full doses. In Ecuador, Pakistan and the Philippines, a higher proportion of MIRS reported being vaccinated compared with MIIS and refugees (p<0.001). MIRS in Nepal reported the lowest level of COVID-19 full vaccination (21.2% (14/66), 95% CI 13.0% to 32.7%). Respondents with formal education not completed had the lowest level of complete vaccination compared with the other education categories (see online supplemental table 5 in Appendix page 5). Among all respondents, 32.4% ((447/1378), 95% CI 30.1% to 35.0%) of individuals received a booster dose, with almost no difference by sex (p=0.470). Booster vaccination was higher among refugees (64.6% (223/345), 95% CI 59.7% to 69.8%) and more widespread in Rwanda (86.3% (177/205), 95% CI 80.9% to 90.4%) (see online supplemental table 6 in Appendix page 6).

Overall, the CVEI was 0.824, suggesting that vaccine coverage was generally higher among migrants compared with the general population globally. However, it is noteworthy that the index exceeded 1 in Ecuador and Pakistan, indicating lower vaccine coverage for refugees and migrants (table 3). According to immigration status, refugees had greater access to vaccines than the general population (CVEI of 0.49). MIRS and MIIS showed CVEI values closer to 1 (0.94 and 0.92, respectively), indicating that they had vaccine coverage similar to that of the general population. In Nepal, only MIRS had less access to the vaccine than the general population, whereas in Ecuador, MIIS had less access to the vaccine. In Rwanda and the Philippines, all categories of migrants had a CVEI of less than 1, meaning that they had more access to vaccination than the general population.

**Table 3:** CVEI by country, gender, migration status, age, duration in the host country, and education

	Overall	Ecuador	Nepal	Pakistan	Philippines	Rwanda
	CVEI	CVEI	CVEI	CVEI	CVEI	CVEI
Gender						
Female	0.78	1.253	0.752	1.068	0.105	0.10
Male	0.86	1.269	0.832	1.235	0.083	0.11
Migration status						
Regular	0.94	0.855	1.333	1.032	0.047	0.17
Irregular	0.92	1.451	0.708	1.369	0	0
Refugee	0.49	1.065	0.486	1.504	0.254	0.09
Age group						
18–29	0.84	1.276	1.296	1.297	0.062	0.106
30–49	0.67	1.125	0.798	1.089	0.091	0.15
50–64	0.91	0.783	1.147	1.113	0.525	0.07
65+	1.03	0.503	0.781	1.144	0	0
Duration in the host country						
< 1 year	0.95	1.684	1.668	0.769	0	0
1–5 years	0.52	1.218	1.311	0.934	0.075	0.19
More than 5 years	0.91	0.814	1.029	1.165	0.109	0.09
Education						
Formal education not completed	1.09	0.646	0.826	1.101	0	0.08
Primary education	1.00	1.574	0.983	1.352	0	0.09
Secondary education completed	0.60	1.176	0.791	1.127	0	0.17
Secondary education not completed	1.0	2.097	1.3	1.376	0	0.1
Technical education	0.52	1.257	0	0	0	0
Bachelor’s degree	0.38	0.973	1.147	0.871	0.116	0.24
Postgraduate degree	0.28	1.131	0	0	0.187	0.23
None of the above	0.80	0	1.254	1.153	0	0.05
I prefer not to answer	1.01	2.715	0	1.386	0	0
Overall	0.824	1.276	0.798	1.148	0.087	0.106

CVEICOVID-19 Vaccine Equity Index

### Barriers

#### Waiting time and distance to vaccination centres

The two most reported difficulties in accessing the COVID-19 vaccine were long waiting times (8.3% (114/1378), 95% CI 6.9% to 9.9%) and vaccination centres being too far away (7.2% (99/1378), 95% CI 5.9% to 8.7%). In the Philippines, 20.8% ((25/120), 95% CI 14.5% to 29.0%) of respondents, and in Ecuador, 13.1% ((45/344), 95% CI 9.9% to 7.1%) of respondents reported long waiting times as the main difficulty, compared with less than 7% in the other countries (p<0.001). In Pakistan, the main difficulty, reported by 12.2% ((61/499), 95% CI 9.6% to 15.4%) of the participants, was the long distance to vaccination centres (see online supplemental table 9 in Appendix page 9).

KIs also mentioned the inaccessibility and inconvenience of vaccination centres (located away from migrants’ and refugees’ sites).

Initially, only select three or four centres were offering vaccinations, and naturally, there would be a lot of rush. Those who would be out to work early morning, it would be a problem as they were unable to afford waiting in line for three or more hours to get vaccinated. (Key informant from Pakistan)

#### Trust

Globally, 37.5% (515/1373) of participants reported little trust (27.6%, 95% CI 25.3% to 30.0%) or no trust (9.9%, 95% CI 8.4% to 11.6%) at all in health providers for COVID-19 vaccines. Overall, men reported higher levels of ‘no trust at all’ than women (16.1% (111/688), 95% CI 13.6% to 19.1% vs 3.7% (25/682), 95% CI 2.5% to 5.4%, p<0.001). However, this was not the case in Nepal and Rwanda. The proportion of respondents who reported little trust (51.3%, 95% CI 46.9% to 55.7%) or no trust (18%, 95% CI 14.9% to 21.7%) at all were the highest in Pakistan and the lowest in Nepal where little trust (7.1%, 95% CI 4.3% to 11.5%) or no trust (1%, 95% CI 0.2% to 3.7%) at all were noted (see online supplemental table 7 in Appendix page 7). Despite this, only 2.7% ((5/184), 95% CI 1.1% to 6.4%) of those who were unvaccinated reported distrust in health authorities’ recommendations as a reason for non-vaccination (see online supplemental table 8 in Appendix page 8).

Most key informants mentioned scepticism towards COVID-19 as a barrier. According to KIs from Pakistan, this scepticism, particularly towards COVID-19 and vaccine effectiveness, appears to be a significant barrier. One key informant from Pakistan explained:

There is some resistance to the vaccine, firstly because people are not clear about COVID-19 itself. Some people say COVID-19 is nothing. Then, there is an ‘undereducated’ population, I wouldn’t say illiterate, but the problem is that they question the reality of COVID. (Key informant from Pakistan)

#### Discrimination and xenophobia

Nine per cent ((126/1378), 95% CI 7.7% to 10.8%) of the respondents witnessed discrimination in the vaccination process, with Pakistan showing the highest level. In this country, the discrimination included instances directed at the respondents themselves (10.6% (53/499), 95% CI 8.2% to 13.6%), as well as observations of discrimination towards other migrants (3.4% (17/499), 95% CI 2.1% to 5.4%) and the public (2.6% (13/499), 95% CI 1.5% to 4.4%). Conversely, in Rwanda, respondents witnessed the least discrimination (2.5% (5/205), 95% CI 1.0% to 5.7%). In all countries, most of the discrimination cases observed were related to the health centre staff, security personnel and the local population (see online supplemental graph 2 in Appendix page 10 and online supplemental tables 10 and 11 in Appendix pages 10 and 11). Among vaccinated respondents, 15.5% ((185/1194), 95% CI 13.5% to 17.7%) said they had perceived differences in how they had been treated compared with most of the population. Overall, this proportion was higher among MIRS (21.2% (104/490), 95% CI 17.8% to 25.1%) than MIIS and refugees (13.9% (53/381), 95% CI 10.8% to 17.8%; and 8.7% (28/323), 95% CI 6.0% to 12.3%, respectively) (p<0.001); however, in Pakistan, 50.0% (41/82) of vaccinated MIIS reported experiencing this difference in treatment (see online supplemental table 12 in Appendix page 11).

Discrimination by health staff or security guards and xenophobia were also reported by some KIs from all countries except Rwanda. In Ecuador, the initial phase of vaccination access was associated with discriminatory practices or perceived discrimination towards migrants, specifically MIIS. This perception in Ecuador changed once vaccines became widely available, and the vaccine coverage for the migrant population became universal in August 2021.

Because when people went alone, there were many times (who said) they didn’t want to attend to me, they said that they couldn’t attend to me without an ID card, or “not with that old mask”, and things like that. So at first I felt that there was this rejection. (Key informant from Ecuador)

Until they had like 100% or a much higher percentage than 80% of the National populations vaccinated, they didn’t start vaccinating foreigners. (Key informant from Ecuador)

#### Lack of identity documentation

Among all the respondents, 6.2% ((85/1378), 95% CI 5.0% to 7.6%) reported being denied access to vaccination due to a lack of identity documentation (IDs). The denial rate was higher among men than women (8.0% (55/691), 95% CI 6.2% to 10.2% vs 4.4% (30/684), 95% CI 3.1% to 6.2%, p<0.006), and also among MIIS than MIRS (7.4% (32/430), 95% CI 5.3% to 10.3% vs 4.5% (27/603), 95% CI 3.1% to 6.5%, p<0.022). However, there was no difference between MIIS and refugees (7.4% (32/430) vs 7.5% (26/345), p<0.480). Rwanda was the country where refugees and migrants were more likely to face vaccination denial due to lack of IDs, with a rate of 11.2% ((23/205), 95% CI 7.6% to 16.3%) (see online supplemental table 13 in Appendix page 11).

This barrier was also mentioned during the qualitative interviews. KIs in Nepal, the Philippines and Pakistan stated the need to present IDs. This barrier was more evident in the initial phase of the vaccination than when the requirement for documentation was abolished. However, according to the KIs, the barrier persisted in some vaccination centres.

In the initial phases, of course, there were some obligations to disclose the age. To disclose the age, they needed an identification card with photo, it was compulsory to bring their migrant card, dentification card with them. But now, vaccines are easily available. They do not need any extra things. (Key informant from Nepal)

#### Timely provision of information

Less than half of the respondents (40.5% (558/1378), 95% CI 37.9% to 43.1%) indicated that timely information was provided. This varied among countries, with the highest percentage reported in Ecuador (51.5% (177/344), 95% CI 46.2% to 56.7%) and the lowest in Rwanda (26.7% (55/205), 95% CI 21.2% to 33.3%) (see online supplemental graph 3 in Appendix page 15). KIs also highlighted barriers related to information provision, such as a lack of knowledge about vaccine effectiveness, language barriers and insufficient information about vaccination points, schemes and campaigns.

The first thing I noticed was that the Hazara people were speaking in their Persian (Farsi) language, and there was not a single Persian-speaking individual in our team. Just imagine. I told the coordinator, you are Pashtu speaking, tell me how many people here speak Pashtu. He said many do. I randomly asked ten women if they can speak Pashtu, four could, six could not. I said what will be done about the six who could not understand Pashtu? (Key informant from Pakistan)

#### Worries about adverse reactions

Among all respondents, 15.3% ((211/1378), 95% CI 13.5% to 17.3%) and 15.5% ((213/1378), 95% CI 13.6% to 17.5%) of respondents were, respectively, very or moderately concerned by possible COVID-19 vaccine reactions (highest in Ecuador with 40.4% (139/344) (25% ((86/344), 95% CI 20.7% to 29.9%) were very concerned and 15.4% ((53/344), 95% CI 12.0% to 19.6%) were moderately concerned) and lowest in Nepal where 10.5% ((22/210), 95% CI 7.0% to 15.4%) were very concerned and 14.3% ((30/210), 95% CI 10.2% to 19.7%) were moderately concerned). In addition, among those who were not vaccinated (mostly from Pakistan), the most reported reason was the risk of adverse reactions to COVID-19 vaccines (33.2% (61/184), 95% CI 26.7% to 40.3%) (see online supplemental table 8 in Appendix page 8). KIs mentioned this barrier for all countries except Rwanda.

Also, a big issue was fear of side effects, that if something were to happen to them where would they go to seek care since they can’t seek treatment from public sector facilities. (Key informant from Pakistan)

### Facilitators

#### Adequate sources and provision of information

Most respondents informed that they received information from various sources, with local news, radio and television being the primary source overall (49.9% (687/1378), 95% CI 47.2% to 52.5%) and particularly in Pakistan (62.1% (310/499), 95% CI 57.8% to 66.3%). Social media was the second most common source overall, reported by 29.2% ((402/1378), 95% CI 26.8% to 31.6%) of respondents. As shown in online supplemental table 14 of the Appendix (page 12), the primary sources of information varied by country, a trend also highlighted by the KIs during the interviews.

In Nepal and Rwanda, participants reported receiving information more frequently from refugee camps’ staff (34.3% (72/210) and 62.9% (129/205), respectively) than from local news, radio and television. In Ecuador and the Philippines, participants primarily accessed COVID-19 vaccine information from the Internet and social media. Qualitative data revealed that authorities in Rwanda used traditional media (newspapers, TV, radio) and refugee camps’ staff to disseminate the information. The findings in Ecuador emphasised that word of mouth was important for refugees and migrants.

We use Local newspapers, radio and TV channels, brochures and billboards, we also use camp staff for every meeting opportunity and use a megaphone in the camp. (Key informant from Rwanda)

A very small percentage of respondents (0.5% overall (7/1378)) reported not receiving information about COVID-19 vaccination. Nevertheless, more than half reported not receiving information promptly.

Regarding the language, KIs from Pakistan, Nepal, Rwanda and the Philippines indicated that local and international government organisations, in coordination with local governments, facilitated the translation of information into different languages to reach the specific migrant and refugee groups in their respective countries. In Ecuador, since the majority of migrants and refugees speak the same language, this was not a significant issue.

#### Targeted vaccination campaigns for refugees and migrants

Qualitative analysis revealed that in most countries, there were vaccination campaigns specifically targeting refugees and migrants and adapted to their needs. These campaigns were coordinated with local governments and local and international organisations. In the Philippines and Ecuador, international organisations contributed to the dissemination of national campaigns, specifically to refugees and migrant groups. KIs from Ecuador and Nepal also mentioned mobile vaccination teams.

In Pakistan, female mobilisers conducted door-to-door vaccinations to increase vaccination among the female population. In addition, the National Command and Operation Centre and provincial and district leaders tried to reach out to refugees and migrants through door-to-door campaigns. However, these specific campaigns were estimated to have reached less than half of the target population.

KIs from Rwanda reported the success of vaccination campaigns, attributing it to a combination of strategies. This included passive communication through media and posters, complemented by outreach information in camps through oral communication by mobile teams.

I think we have reached more than 80 % of all migrants and refugees. We used Local newspapers, radio and TV channels, brochures and billboards. We also used camp staff for every meeting opportunity and used a megaphone in the camp. (Key informant from Rwanda)

Globally, 64.2% ((884/1378), 95% CI 61.6% to 66.6%) of respondents believed that government campaigns were successful. In Rwanda, 81.5% ((167/205), 95% CI 75.5% to 86.2%) of respondents thought their campaigns were successful, while in Pakistan only 45.3% ((226/499), 95% CI 41.0% to 49.7%) of respondents felt similarly. The sentiment was evenly distributed among men and women (64.3% (444/691) vs 64.0% (438/684)) but varied more among MIRS (56.9% (343/603)) compared with MIIS and refugees (69.1% (297/430) and 70.7% (244/345), respectively, with p<0.001). In Pakistan, refugees were significantly more sceptical about the success of the campaigns compared with other migrants (p<0.01). A similar, though not statistically significant, trend was noted in Ecuador. In contrast, in Nepal and Rwanda, MIRS were more likely to reject the success of the government campaigns than MIIS and refugees, though this variation lacked statistical significance (see online supplemental table 15 in Appendix page 13).

#### Distribution of vaccination centres

Among all respondents, 68.9% ((950/1378), 95% CI 66.4% to 71.3%) reported that the health centres set up at the national level were accessible in their communities. This perception, evenly distributed across genders, was lower among MIRS (57.2% (345/603), 95% CI 53.2% to 61.1%) compared with MIIS and refugees (78.6% (338/430), 95% CI 74.5% to 82.2%; and 77.4% (267/345), 95% CI 72.7% to 81.5%, p<0.001). In most countries, at least 69.5% of the respondents believed that the health centres set up for COVID-19 vaccination were accessible to their community. However, only 44.3% ((221/499), 95% CI 40.0% to 48.7%) of refugees and migrants from Pakistan shared this perception (p<0.002) (see online supplemental table 16 in Appendix page 14).

Additionally, 45.9% ((632/1378), 95% CI 43.2% to 48.5%) of all respondents reported placing vaccination centres in different areas of the city as a facilitator (see online supplemental graph 3 in Appendix page 15). This percentage was notably higher in Rwanda (68.8% (141/205), 95% CI 62.1% to 74.8%) and Ecuador (64.8% (223/344), 95% CI 59.6% to 69.7%) compared with the other countries (p<0.001). KIs from Ecuador, Nepal and Rwanda also cited fixed vaccination centres as facilitators.

#### Political commitment

Qualitative analysis, including policy analysis, also showed that national policies in all countries were very explicit on the obligation to include all citizens and residents in national health plans, including vaccination programmes. For instance, the Ecuadorian vaccination programme 9/1000, launched at the beginning of the Guillermo Lasso government on 24 May 2021, aimed to vaccinate 9 million people within the first 1000 days of his term and was the first in the region to make vaccines available to its migrant population. In Rwanda, KIs highlighted strong political will, meaning government commitment and support, as an enabling factor to inclusive and equitable policies by listing refugees and migrants among priority populations.

I can say that there is a strong political will to get everyone vaccinated and the service is free for everyone. All the key materials were provided in languages of the refugees like Amharic, Arab, French Kirundi … People with comorbidity conditions were prioritized. There are no movement restrictions on refugees and everything we do regarding refugee camps; we do it in a comprehensive way with the hosting communities. Everyone, refugee communities and hosting communities has access to the same information. (Key informant from Rwanda)

In most countries, the COVID-19 vaccine was offered entirely free of charge. In contrast, in Pakistan, 5.5% ((19/348), 95% CI 3.5% to 8.4%) of vaccinated respondents reported paying for appointments (p<0.001) and 2.3% ((8/348), 95% CI 1.2% to 4.5%) paid for the vaccination certificate (p<0.001) (see online supplemental table 17 in Appendix page 15). KIs in Ecuador, Nepal and Rwanda also mentioned free access as a facilitator.

In Nepal, the coordination and collaboration between the central, provincial and local governments and NGOs were reported to be a catalyst for managing COVID-19 cases and high vaccination coverage. In addition, Ecuador reported the introduction of a single-dose vaccine for migrants in mobility. One KI from the Philippines mentioned:

Policies are inclusive. We made a deliberate policy when we were defining coverage of the national vaccination program under the principle that these are people within our midst, and so it makes sense that they have to be included. They are exposed as we are with the virus. There are two purposes of the vaccination: to protect the person from illness and death and to prevent the transmission of the virus. Everybody needs protection. (Key informant from the Philippines)

Despite the inclusive policies, the KIs said these only sometimes translated into inclusive services, particularly at the start of the vaccination programmes. In Ecuador, Nepal and Pakistan, refugees and migrants were not included in the first wave of vaccination mainly due to the registration mechanism that implicitly excluded these groups, such as the use of election cards in Ecuador and the national database through National Database and Registration Authority in Pakistan. After these initial challenges, the registration process for vaccination was simplified. In Nepal and Ecuador, proactive migrant activities were organised after the general population was sufficiently covered. Community representatives in Pakistan reported instances where COVID-19 vaccination cards were sold to some migrant groups who did not want to be vaccinated to avoid registration by national authorities.

#### Other facilitators

Among those who received the COVID-19 vaccine, a substantial 62.1% ((741/1194), 95% CI 59.3% to 64.8%) stated that their motivation to receive the COVID-19 vaccine was to protect others, including their family, friends and the community. In addition, 60.7% ((725/1194), 95% CI 57.9% to 63.5%) cited the sense of duty to vaccinate (see online supplemental table 18 in Appendix page 15).

KIs from Pakistan and the Philippines reported the provision of incentives as facilitators.

During October 2021, in the beginning of vaccine campaigns we faced a lot of resistance from them but when they were facilitated with some basic medicines for different diseases, which created a bond of trust, they were coming happily to vaccine centres seeking COVID-19 vaccination. (Key informant from Pakistan)

Among vaccinated respondents, 59.4% ((709/1194), 95% CI 56.6% to 62.1%) reported the vaccine requirement for entry into establishments as their reason for getting vaccinated. This was reported as the main reason for vaccination in the Philippines (86.4% (102/118), 95% CI 79.0% to 91.5%). This observation was also mentioned by KIs from Pakistan and the Philippines, emphasising that a national mandate for vaccination cards to access public places enhanced the willingness of refugees and migrants to get vaccinated, given they had no alternative if they wished to access those locations. In Pakistan, it was also noted that mandatory vaccination requirements by schools served as another facilitator.

Trust and a positive narrative in terms of acceptance of migrants also played a role in facilitating their access to vaccines, as reported in Nepal and Pakistan.

## Discussion

This multicountry, mixed-method study aimed to estimate the proportion of COVID-19 vaccination coverage among refugees and migrants and to identify the barriers and facilitators related to the vaccination process. The five countries completed the qualitative and the quantitative phases, and the survey reached the targeted sample size in each country.

Overall, the COVID-19 vaccine coverage of refugees and migrants, including MIIS, was high. Our study found that 86.6% of the survey respondents had been vaccinated with at least one dose and 67.5% had received full doses. In addition, the global-level equity index suggests that respondents had greater access to vaccination compared with the general population. Nevertheless, in Ecuador and the Philippines, refugees and migrants faced lower access to vaccination than the general population. The overall positive findings for refugees and migrants differ from published data on COVID-19 vaccine coverage among some migrant groups in European countries.^30^ Moreover, they contrast with data from Our World in Data, which reported a single-dose coverage of approximately 60% and full-dose coverage of 54.2% in low- and middle-income countries (LMICs) by mid-2022, the same period as our study.^31^ Nonetheless, our results align with other studies indicating an average COVID-19 vaccine acceptance rate of 80.3% in LMICs.^13^

Our qualitative data showed heterogeneity in the rollout of refugee and migrant-specific vaccination initiatives within our study countries. These initiatives were either integrated at the start of the nationwide vaccination programme, as observed in Rwanda, or implemented later, following the initial administration of COVID-19 vaccines to the general population, as seen in Ecuador and Pakistan. Within our study countries, a range of policies and administrative and legal factors were elucidated, enabling or restricting access to COVID-19 vaccines for refugees and migrants.^11 32^ Notably, most study countries offered COVID-19 services without charge, deflecting direct financial burden as a barrier. This contrasts with pre-COVID-19 studies in other settings, including some high-income countries, where migrants must bear direct vaccination expenses, depending on their immigration status.^33^

Overall, some main barriers cited by the survey participants encompassed extended waiting times at vaccination centres and the considerable distance to vaccination sites, particularly in Pakistan and the Philippines. The cost of vaccination emerged as a less frequently reported barrier within our study countries. This finding diverges from reports from organisations that identified the lack of clarity regarding COVID-19 vaccination fees for non-citizens or undocumented people not enrolled in national health insurance schemes as an obstacle for migrants.^34^ Prior research also showed that low knowledge among healthcare professionals regarding migrant health needs, eligibility criteria, language barriers and a lack of empathy or cultural competency could result in migrants being denied vaccinations regardless of their policy-based entitlement.^35 36^ During our initial interviews, the KIs also mentioned these barriers; however, in our survey, less than 10% of migrants reported experiencing or witnessing discrimination by healthcare staff, security personnel or the general population.

In addition, many studies have underscored the prevalence of mistrust and hesitancy towards COVID-19 vaccination, attributed to concerns arising from the dissemination of misleading COVID-19 vaccination information.^37^ Around two-fifths of our respondents indicated little or no trust. Globally, this elevated level of distrust is particularly prominent among men compared with women. The same trend is also found in most countries. Several studies show that the degree of trust in immunisation, health services and providers can be a critical factor in how refugee and migrant populations perceive vaccination, subsequently impacting vaccine uptake.^1438^,^40^

Regarding facilitators, the main factor cited globally was the decentralisation of vaccination services. This factor was noted in Ecuador and Rwanda. In Ecuador, 63.9% of respondents declared they were not living far from vaccination centres. Additionally, Ecuador stood out as the country where more than half of the respondents thought timely information was provided, while only one out of four agreed with this statement in Rwanda. Our findings are consistent with those of a published literature review that identified several important barriers and facilitators influencing COVID-19 vaccination uptake among refugees and migrants.^30 41 42^

While our study provides valuable insights to inform policy recommendations and enhance vaccine uptake among refugees and migrants, several practical challenges must be acknowledged. Simplifying the vaccination registration process can be hindered by bureaucratic procedures. Designing and implementing targeted campaigns require addressing diverse cultural and linguistic needs. Deploying mobile vaccination teams presents logistical challenges such as funding and vaccine storage. Building trust among refugees and migrants who may have experienced past traumas or distrust towards authorities is a complex process. Combatting misinformation and vaccine hesitancy demands continuous effort and reliable information sources. By recognising these challenges, policymakers can better implement recommendations to ensure equitable vaccine access and health service delivery.

### Limitations and strengths

Our study has limitations. The main one pertains to the differences between the diverse methods used for participant recruitment. Specifically, reaching MIIS required a distinct approach, necessitating a snowball sampling method. This technique can result in a non-representative sample, introducing the possibility that our MIIS sample may not accurately reflect the broader MIIS population. This limitation should be considered when interpreting the findings, as it may impact the generalisability of our results. Additionally, the nature of the target population, comprising regular and irregular migrants, as well as refugees, did not permit the use of a sampling frame for selecting respondents. This limitation affects the representativeness of the sample and prevents us from conducting inferential analysis at any level.

Second, the main target group was not stratified by key characteristics, such as gender and migration status. The absence of stratified analysis could mask important variations within the sample, potentially leading to an incomplete understanding of the differences between various subgroups. Consequently, MIRS in Rwanda were inadequately represented in the sample.

Third, the reliance on self-reported information, including vaccination status, poses a risk of recall bias or information concealment, which can lead to overestimation or inaccuracies in the reported data. Due to the nature and design of the study, we were unable to verify the self-reported data against official verification records.

Fourth, another limitation stems from the absence of aggregated COVID-19 vaccination prevalence data for the countries combined, as they do not form a unified entity. To remedy this situation, we used the mean prevalence derived from individual country prevalences, weighted by their respective population sizes.

Additionally, our study was conducted in different phases during a rapidly changing health crisis. Although the short timeframe between phases I and II of our study helped mitigate some of the issues, differences in vaccine availability between the KIs interviews and FGDs and the survey period could potentially have impacted our findings. This may have led to variations between our quantitative and qualitative analyses, with the qualitative feedback generally being more critical of the vaccine rollout compared with the quantitative results.

Furthermore, the calculation of the CVEI was based on comparing the proportion of unvaccinated individuals in subgroups to the total population. This method assumes reliable data collection and representativeness of the sampled populations. However, variations in data reporting and potential sampling bias may affect the accuracy of the CVEI. Despite these challenges, the CVEI provides a valuable measure of vaccination equity, highlighting disparities that need to be addressed.

Our study has notable strengths. The data collection process yielded an adequate sample size for analyses and included the challenging-to-reach MIIS group. Additionally, to our knowledge, this study stands out as the first multicountry mixed-method study specifically evaluating COVID-19 vaccination coverage among refugees and migrants.

## Conclusion

Our study results indicate no inequities in vaccination coverage between MIRS, MIIS and the general population at the global level. Additionally, refugees had greater access to vaccines than the general population in most participating countries. However, we should be cautious with these results, as our survey was conducted in mid-2022 and did not capture the early delays or population group differences in vaccine distribution at the start of the vaccination campaigns. The KIs reported such differences, which are widely documented in previous COVID-19 literature. In addition, the effectiveness of specific refugee and migrant vaccination campaigns varied, as seen in Pakistan, where efforts were unsuccessful.

Future research should focus on identifying and addressing barriers such as long wait times, distance to vaccination sites and trust issues. Targeted strategies like simplified registration processes, targeted campaigns and mobile vaccination teams can enhance vaccine uptake. Building trust with host country authorities and healthcare providers is also crucial to improve vaccine acceptance among refugees and migrants.

Despite limitations such as diverse recruitment methods and reliance on self-reported data, our study provides valuable insights into vaccine coverage among refugees and migrants. Addressing these challenges is essential for future vaccine deployments and health service delivery, helping to build more resilient and inclusive health systems for populations in vulnerable situations, particularly during health crises like the pandemic.

## supplementary material

10.1136/bmjopen-2024-087629 online supplemental file 1

10.1136/bmjopen-2024-087629 online supplemental file 2

10.1136/bmjopen-2024-087629 online supplemental file 3

## Data Availability

Data are available upon reasonable request.
