# Supplementary material for: Deployment and uptake of COVID-19 vaccines for refugees and migrants in regular and irregular situations: a mixed-method multicountry study
Source: BMJ Open. 2025 Feb 2;15(1):e087629. doi: 10.1136/bmjopen-2024-087629 (PMC11904359; doi:10.1136/bmjopen-2024-087629)
Supplement: online supplemental file 1 [file bmjopen-15-1-s001.docx]

APPENDIX

**Figure 1: Description of the study phases, the related conceptual frameworks and the expected outcomes**


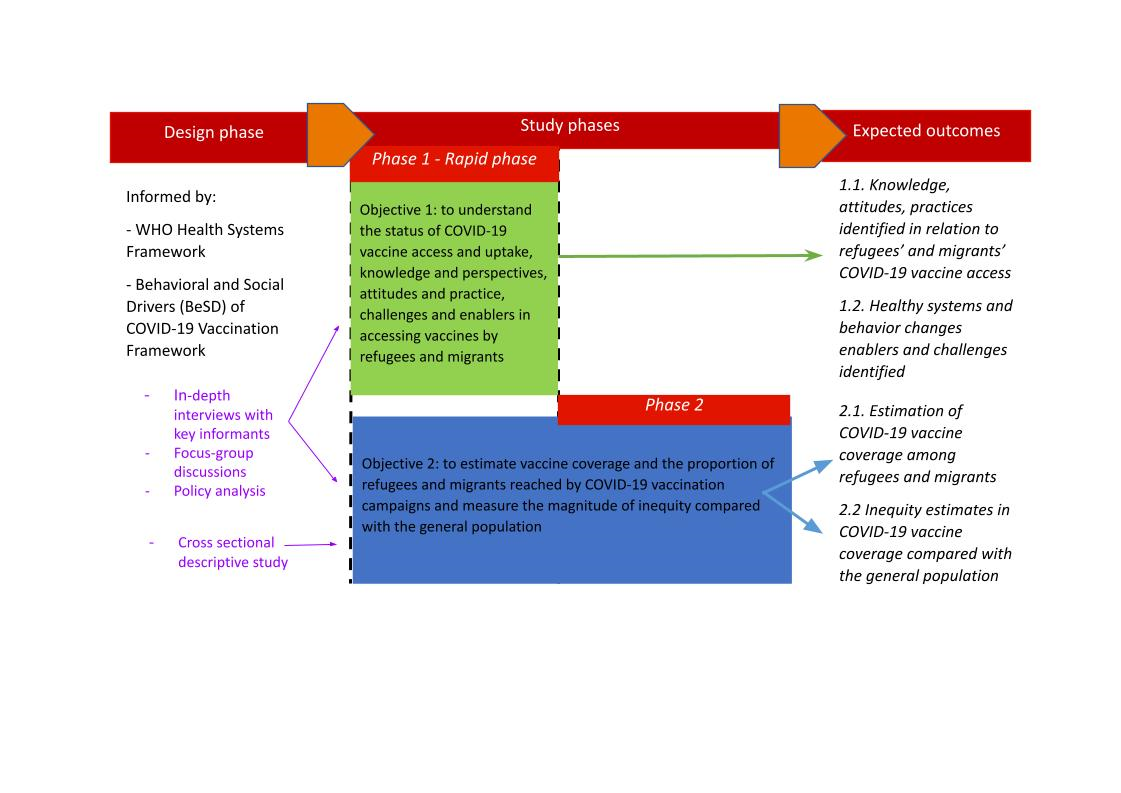


**Figure 2. The World Health Organization (WHO) Health Systems Framework**


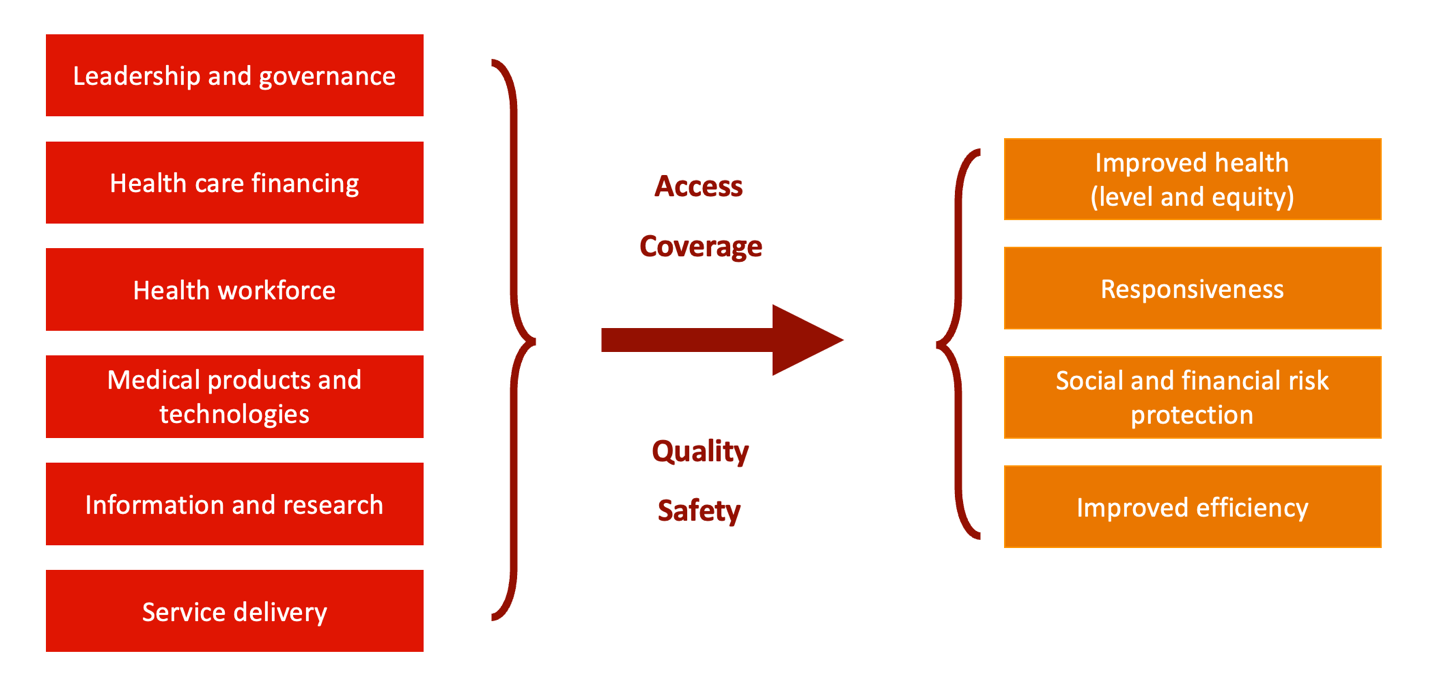


**Figure 3. Behavioural and Social Drivers (BeSD) of COVID-19 Vaccination Framework**

***
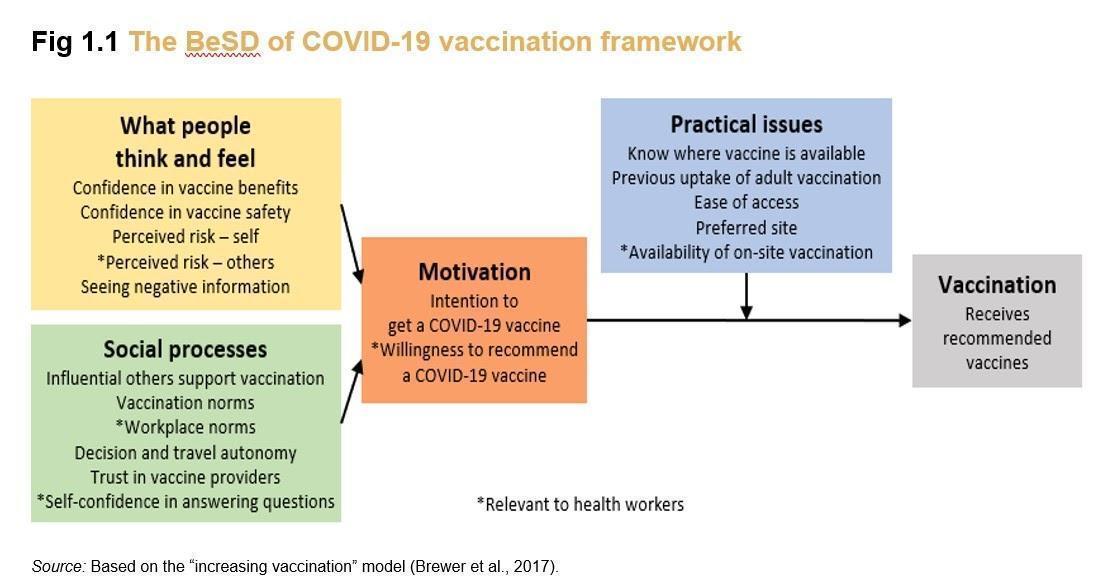
***

**Table 1. General, refugee, and migrant populations and COVID-19 coverage in the five study countries as of 30 March 2022**

| **Study countries**  **Country** | **WHO region** | **Total country population** | **Estimated migrant population** | **Estimated refugee population** | **% of total population fully vaccinated against COVID-19** |
| --- | --- | --- | --- | --- | --- |
| **Ecuador** | PAHO | 18,000,000 | 784,787 | 503,644 | 79% |
| **Nepal** | SEARO | 28,000,000 | 487,564 | 19,634 | 66% |
| **Pakistan** | EMRO | 227,000,000 | 3,276,580 | 1,428,147 | 47% |
| **Philippines** | WPRO | 109,000,000 | 225,525 | 1,023 | 61% |
| **Rwanda** | AFRO | 12,950,000 | 513,907 | 145,552 | 64% |

Sources: World Bank (total population data). United Nations Department of Economic and Social Affairs, Population Division. International Migrant Stock 2020 (migrant population data). UNHCR Refugee Data Finder 2021 (refugee and asylum-seeker population data). Our World in Data (COVID-19 vaccination data, fully vaccinated defined as 1 dose of Johnson & Johnson vaccine or 2 doses of all other available vaccines).

**Table 2. List of selected countries, research partners and lead researchers**

| **Country** | **National research institutes** | **Lead researchers** |
| --- | --- | --- |
| **Ecuador** | **(a)**Universidad San Francisco de Quito, and (b) Universidad Central del Ecuador | (a)Enrique Terán, Cheryl Martens, (b) Maria Belen Mena, Lorena Castellanos |
| **Nepal** | Kathmandu University School of Medical Sciences | Biraj Karmacharya, Prasanna Rai, Anjali Joshi, Sabina Marasini |
| **Pakistan** | Aga Khan University | Jai Das |
| **The Philippines** | The Demographic Research and Development Foundation, Inc. | Vincente B. Jurlano, Nimfa B. Ogena, Maria Midea M. Kabamalan, Gilda Salvacion Diaz |
| **Rwanda** | University of Rwanda | Laetitia Nyirazinyoye |

**Graph 1. Distribution of high-risk conditions by country**


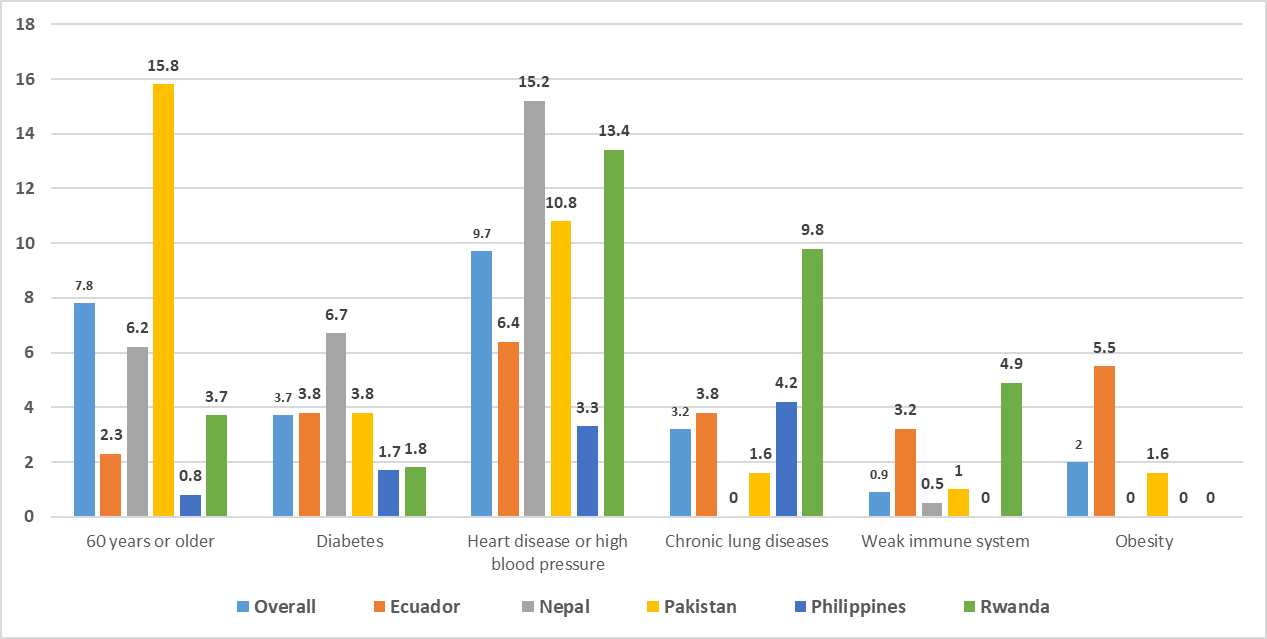


**Table 3. Frequency and proportion of respondents who reported to have been infected by country, gender, migration status, age, and duration in the host country**

|  | Overall (n=1378) | | Ecuador (n=344) | | Nepal   (n=210) | | Pakistan (n=499) | | Philippines (n=120) | | Rwanda (205) | |
| --- | --- | --- | --- | --- | --- | --- | --- | --- | --- | --- | --- | --- |
|  | Freq. | % | Freq. | % | Freq. | % | Freq. | % | Freq. | % | Freq. | % |
| ***Gender*** |  |  |  |  |  |  |  |  |  |  |  |  |
| Female | 96 | 14.0 | 51 | 28.8 | 15 | 16.7 | 10 | 3.4 | 10 | 40 | 10 | 7.6 |
| Male | 114 | 16.5 | 62 | 37.8 | 12 | 10 | 15 | 6.3 | 23 | 24.2 | 2 | 2.8 |
| Other | 2 | 66.7 | 2 | 66.7 | 0 | 0.0 | 0 | 0.0 | 0 | 0.0 | 0 | 0.0 |
| ***Migration status*** |  |  |  |  |  |  |  |  |  |  |  |  |
| Regular | 68 | 11.3 | 30 | 33.3 | 1 | 1.5 | 19 | 5.5 | 15 | 26.8 | 3 | 6.7 |
| Irregular | 90 | 20.9 | 79 | 33.3 | 0 | 0.0 | 3 | 2.7 | 8 | 24.2 | 0 | 0.0 |
| Refugee | 54 | 15.7 | 6 | 35.3 | 26 | 25.7 | 3 | 7.1 | 10 | 32.3 | 9 | 5.8 |
| ***Age group*** |  |  |  |  |  |  |  |  |  |  |  |  |
| 18 – 29 | 68 | 19.2 | 35 | 30.7 | 3 | 6.5 | 7 | 8.3 | 22 | 25.9 | 1 | 4.2 |
| 30 – 49 | 90 | 17.9 | 62 | 36.7 | 7 | 10.8 | 6 | 5.2 | 10 | 34.5 | 5 | 4 |
| 50 – 64 | 32 | 11.1 | 15 | 28.8 | 8 | 15.4 | 3 | 2.1 | 1 | 20.0 | 5 | 13.2 |
| 65 or more | 22 | 9.4 | 3 | 33.3 | 9 | 19.1 | 9 | 5.7 | 0 | 0.0 | 1 | 5.6 |
| ***Duration in the host country*** |  |  |  |  |  |  |  |  |  |  |  |  |
| < 1 year | 22 | 18.8 | 22 | 25.6 | 0 | 0.0 | 0 | 0.0 | 0 | 0.0 | 0 | 0.0 |
| 1 – 5 years | 89 | 27.1 | 66 | 31.7 | 0 | 0 | 1 | 14.3 | 21 | 30 | 1 | 3.7 |
| More than 5 years | 101 | 10.8 | 27 | 54 | 27 | 14.6 | 24 | 5.1 | 12 | 25 | 11 | 6.3 |
| **Total** | 212 | 15.4 | 115 | 33.4 | 27 | 12.9 | 25 | 5.0 | 33 | 27.5 | 12 | 5.9 |

**Table 4. Frequency and proportion of vaccinated against COVID-19 by country, gender, migration status, age, duration in the host country, and education**

|  | Overall   (n=1378) | | Ecuador (n=344) | | Nepal (n=210) | | Pakistan (n=499) | | Philippines (n=120) | | Rwanda (n=205) | |
| --- | --- | --- | --- | --- | --- | --- | --- | --- | --- | --- | --- | --- |
|  | Freq | % [CI] | Freq | % | Freq | % | Freq | % | Freq | % | Freq | % |
| ***Gender*** |  |  |  |  |  |  |  |  |  |  |  |  |
| Female | 573 | 83.8[80.8-86.4] | 171 | 96.6 | 86 | 95.6 | 160 | 61.8 | 25 | 100 | 131 | 98.5 |
| Male | 618 | 89.4[86.9-91.5] | 149 | 90.9 | 116 | 96.7 | 188 | 78.3 | 93 | 97.9 | 72 | 100 |
| Other | 3 | 100[100- 100] | 3 | 100 | 0 | 0.0 | 0 | 0.0 | 0 | 0.0 | 0 | 0.0 |
| ***Migration status*** |  |  |  |  |  |  |  |  |  |  |  |  |
| Regular | 490 | 81.3[82.1-84.2] | 88 | 97.8 | 61 | 92.4 | 239 | 69.5 | 56 | 100 | 46 | 97.9 |
| Irregular | 381 | 88.6[85.2-91.3] | 220 | 92.8 | 42 | 97.7 | 82 | 72.6 | 33 | 100 | 4 | 100 |
| Refugee | 323 | 93.6[90.5-95.8] | 15 | 88.2 | 99 | 98.0 | 27 | 64.3 | 29 | 93.5 | 153 | 99.4 |
| ***Age group*** |  |  |  |  |  |  |  |  |  |  |  |  |
| 18 – 29 | 316 | 89.3[82.1-90.4] | 105 | 92.1 | 44 | 95.7 | 58 | 69 | 84 | 98.8 | 25 | 100 |
| 30 – 49 | 460 | 91.5[89.0-93.9] | 160 | 94.7 | 62 | 95.4 | 88 | 75.9 | 28 | 96.6 | 122 | 98.4 |
| 50 – 64 | 241 | 83.7[73.0-87.7] | 49 | 94.2 | 49 | 94.2 | 100 | 70.9 | 5 | 100 | 38 | 100 |
| 65 or more | 177 | 76.4[71.1-81.9] | 9 | 100 | 47 | 100 | 102 | 64.6 | 1 | 100 | 18 | 100 |
| ***Duration in the host country*** |  |  |  |  |  |  |  |  |  |  |  |  |
| < 1 year | 108 | 92.3[85.9-95.9] | 80 | 93 | 10 | 90.9 | 15 | 88.2 | 2 | 100 | 1 | 100 |
| 1 – 5 years | 311 | 94.8[91.8-96.7] | 195 | 93.8 | 13 | 92.9 | 6 | 85.7 | 69 | 98.6 | 27 | 96.4 |
| More than 5 years | 775 | 83.1[80.5-85.3] | 48 | 96 | 179 | 96.8 | 327 | 68.8 | 47 | 97.9 | 174 | 99.4 |
| ***Education*** |  |  |  |  |  |  |  |  |  |  |  |  |
| Formal education not completed | 310 | 72.8[68.3-76.8] | 6 | 85.7 | 25 | 100 | 245 | 68.1 | 0 | 0.0 | 34 | 100 |
| Primary education | 131 | 86.2[79.7-90.8] | 23 | 100 | 40 | 95.2 | 39 | 67.2 | 0 | 0.0 | 29 | 100 |
| Secondary education completed | 220 | 93.6[89.7-96.1] | 118 | 92.9 | 29 | 100 | 23 | 79.3 | 35 | 100 | 15 | 100 |
| Secondary education not completed | 111 | 94.1[88.1-97.1] | 36 | 87.8 | 30 | 100 | 17 | 89.5 | 2 | 100 | 26 | 100 |
| Technical education | 23 | 100[100-100] | 18 | 100 | 0 | 0 | 0 | 0 | 1 | 100 | 4 | 100 |
| Bachelor degree | 188 | 95.4[91.4-97.6] | 89 | 95.7 | 7 | 87.5 | 4 | 80 | 66 | 97.1 | 22 | 95.7 |
| Postgraduate degree | 34 | 97.1[82.2-99.6] | 7 | 87.5 | 1 | 100 | 0 | 0.0 | 14 | 100 | 12 | 100 |
| None of the above | 142 | 92.2[86.7-95.5] | 12 | 100 | 70 | 93.3 | 11 | 64.7 | 0 | 0.0 | 49 | 98 |
| I prefer not to answer | 35 | 92.1[78.2.0-97.4] | 14 | 93.3 | 0 | 0.0 | 9 | 81.8 | 0 | 0.0 | 12 | 100 |
| **Total** | **1,194** | **86.6[84.7-88.3]** | **323** | **93.9 [91.4-96.4]** | **202** | **96.2**  **[93.6-98.8]** | **348** | **69.7 [65.5-73.6]** | **118** | **98.3 [91.4-96.4]** | **203** | **99 [97.7-100]** |

**Table 5. Frequency and proportion receiving full COVID-19 vaccine series by country, gender, migration status, age, duration in the host country, and education**

|  | Overall (n=1378) | | Ecuador (n=344) | | Nepal (n=210) | | Pakistan (n=499) | | Philippines (n=120) | | Rwanda   (n=205) | |
| --- | --- | --- | --- | --- | --- | --- | --- | --- | --- | --- | --- | --- |
|  | Freq. | % | Freq. | % | Freq. | % | Freq. | % | Freq. | % | Freq. | % |
| ***Gender*** |  |  |  |  |  |  |  |  |  |  |  |  |
| Female | 462 | 67.5 | 128 | 72.3 | 50 | 58.1 | 132 | 51.0 | 24 | 96.0 | 128 | 96.2 |
| Male | 444 | 64.3 | 118 | 72.0 | 61 | 52.6 | 104 | 43.3 | 92 | 96.8 | 69 | 95.8 |
| Other | 1 | 33.3 | 1 | 33.3 | 0 | 0.0 | 0 | 0.0 | 0 | 0.0 | 0 | 0.0 |
| ***Migration status*** |  |  |  |  |  |  |  |  |  |  |  |  |
| Regular | 367 | 60.9 | 73 | 81.1 | 14 | 23 | 181 | 52.6 | 55 | 98.2 | 44 | 93.6 |
| Irregular | 265 | 61.6 | 161 | 67.9 | 25 | 59.5 | 42 | 37.2 | 33 | 100.0 | 4 | 100.0 |
| Refugee | 275 | 79.7 | 13 | 76.5 | 72 | 72.7 | 13 | 31.0 | 28 | 90.3 | 149 | 96.8 |
| ***Age group*** |  |  |  |  |  |  |  |  |  |  |  |  |
| 18 – 29 | 231 | 65.3 | 69 | 60.5 | 20 | 45.5 | 34 | 40.5 | 83 | 97.6 | 25 | 100.0 |
| 30 – 49 | 364 | 72.4 | 127 | 75.1 | 34 | 54.8 | 58 | 50.0 | 28 | 96.6 | 117 | 94.4 |
| 50 – 64 | 179 | 62.2 | 43 | 82.7 | 26 | 53.1 | 69 | 48.9 | 4 | 80.0 | 37 | 97.4 |
| 65 or more | 133 | 57.1 | 8 | 88.9 | 31 | 66 | 75 | 47.5 | 1 | 100.0 | 18 | 100.0 |
| ***Duration in the host country*** |  |  |  |  |  |  |  |  |  |  |  |  |
| < 1 year | 71 | 60.7 | 54 | 62.8 | 3 | 30 | 11 | 64.7 | 2 | 100.0 | 1 | 100.0 |
| 1 – 5 years | 257 | 78.4 | 152 | 73.1 | 6 | 46.2 | 4 | 57.1 | 2 | 2.9 | 26 | 92.9 |
| More than 5 years | 579 | 62.1 | 41 | 82.0 | 102 | 57 | 221 | 46.5 | 46 | 95.8 | 169 | 96.6 |
| ***Education*** |  |  |  |  |  |  |  |  |  |  |  |  |
| Formal education not completed | 233 | 54.7 | 6 | 85.7 | 16 | 64.0 | 178 | 49.4 | 0 | 0.0 | 33 | 97.1 |
| Primary education | 89 | 58.6 | 15 | 65.2 | 24 | 60.0 | 22 | 37.9 | 0 | 0.0 | 28 | 96.6 |
| Secondary education completed | 176 | 74.9 | 94 | 74.0 | 19 | 65.5 | 14 | 48.3 | 35 | 100.0 | 14 | 87.5 |
| Secondary education not completed | 69 | 58.5 | 22 | 53.7 | 13 | 43.3 | 7 | 36.8 | 2 | 100.0 | 25 | 96.2 |
| Technical education | 18 | 78.3 | 13 | 72.2 | 0 | 0.0 | 0 | 0.0 | 1 | 100.0 | 4 | 100.0 |
| Bachelor degree | 166 | 84.3 | 73 | 78.5 | 4 | 57.1 | 3 | 60.0 | 65 | 95.6 | 21 | 91.3 |
| Postgraduate degree | 31 | 88.6 | 6 | 75.0 | 1 | 100 | 0 | 0.0 | 13 | 92.9 | 11 | 91.7 |
| None of the above | 103 | 66.9 | 12 | 100.0 | 34 | 48.6 | 8 | 47.1 | 0 | 0.0 | 49 | 98.0 |
| I prefer not to answer | 22 | 57.9 | 6 | 42.9 | 0 | 0.0 | 4 | 36.4 | 0 | 0.0 | 12 | 100.0 |
| **Total** | **907** | **65.8** | **247** | **71.8** | **111** | **52.9** | **236** | **47.3** | **116** | **96.7** | **197** | **96.1** |

**Table 6. Frequency and proportion of booster vaccination by country, gender, immigration status, age, duration in the host country, and education**

|  | Overall (n=1378) | | Ecuador (n=344) | | Nepal (n=210) | | Pakistan (n=499) | | Philippines (n=120) | | Rwanda   (n=205) | |
| --- | --- | --- | --- | --- | --- | --- | --- | --- | --- | --- | --- | --- |
|  | Freq. | % | Freq. | % | Freq. | % | Freq. | % | Freq. | % | Freq. | % |
| ***Gender*** |  |  |  |  |  |  |  |  |  |  |  |  |
| Female | 225 | 32.9 | 43 | 24.3 | 50 | 55.6 | 7 | 2.7 | 11 | 44.0 | 114 | 85.7 |
| Male | 222 | 32.1 | 50 | 30.5 | 59 | 49.2 | 5 | 2.1 | 46 | 48.4 | 62 | 86.1 |
| Other | 0 | 0.0 | 0 | 0.0 | 0 | 0.0 | 0 | 0.0 | 0 | 0.0 | 0 | 0.0 |
| ***Migration status*** |  |  |  |  |  |  |  |  |  |  |  |  |
| Regular | 120 | 19.9 | 28 | 31.1 | 14 | 21.2 | 8 | 2.3 | 31 | 55.4 | 39 | 83.0 |
| Irregular | 104 | 24.2 | 60 | 25.3 | 23 | 53.5 | 4 | 3.5 | 15 | 45.5 | 2 | 50.0 |
| Refugee | 223 | 64.6 | 5 | 29.4 | 72 | 71.3 | 0 | 0.0 | 11 | 35.5 | 135 | 87.7 |
| ***Age group*** |  |  |  |  |  |  |  |  |  |  |  |  |
| 18 – 29 | 102 | 28.8 | 21 | 18.4 | 20 | 43.5 | 0 | 0.0 | 42 | 49.4 | 19 | 76.0 |
| 30 – 49 | 196 | 39.0 | 44 | 26.0 | 32 | 49.2 | 2 | 1.7 | 13 | 44.8 | 105 | 84.7 |
| 50 – 64 | 89 | 30.9 | 23 | 44.2 | 26 | 50.0 | 4 | 2.8 | 1 | 20.0 | 35 | 92.1 |
| 65 or more | 60 | 25.8 | 5 | 55.6 | 31 | 66.0 | 6 | 3.8 | 1 | 100.0 | 17 | 94.4 |
| ***Duration in the host country*** |  |  |  |  |  |  |  |  |  |  |  |  |
| < 1 year | 23 | 19.7 | 17 | 19.8 | 3 | 27.3 | 1 | 5.9 | 1 | 50.0 | 1 | 100.0 |
| 1 – 5 years | 124 | 37.8 | 57 | 27.4 | 6 | 42.9 | 0 | 0.0 | 36 | 51.4 | 25 | 89.3 |
| More than 5 years | 300 | 32.2 | 19 | 38.0 | 100 | 54.1 | 11 | 2.3 | 20 | 41.7 | 150 | 85.7 |
| ***Education*** |  |  |  |  |  |  |  |  |  |  |  |  |
| Formal education not completed | 55 | 12.9 | 2 | 28.6 | 15 | 60.0 | 9 | 2.5 | 0 | 0.0 | 29 | 85.3 |
| Primary education | 54 | 35.5 | 5 | 21.7 | 23 | 54.8 | 1 | 1.7 | 0 | 0.0 | 25 | 86.2 |
| Secondary education completed | 67 | 28.5 | 27 | 21.3 | 19 | 65.5 | 0 | 0.0 | 13 | 37.1 | 8 | 50.0 |
| Secondary education not completed | 45 | 38.1 | 8 | 19.5 | 13 | 43.3 | 0 | 0.0 | 1 | 50.0 | 23 | 88.5 |
| Technical education | 10 | 43.5 | 5 | 27.8 | 0 | 0.0 | 0 | 0.0 | 1 | 100.0 | 4 | 100.0 |
| Bachelor degree | 92 | 46.7 | 33 | 35.5 | 4 | 50.0 | 1 | 20.0 | 35 | 51.5 | 19 | 82.6 |
| Postgraduate degree | 21 | 60.0 | 4 | 50.0 | 1 | 100.0 | 0 | 0.0 | 7 | 50.0 | 9 | 75.0 |
| None of the above | 85 | 55.2 | 3 | 25.0 | 34 | 45.3 | 1 | 5.9 | 0 | 0.0 | 47 | 94.0 |
| I prefer not to answer | 18 | 47.4 | 6 | 40.0 | 0 | 0.0 | 0 | 0.0 | 0 | 0.0 | 12 | 100.0 |
| **Total** | **447** | **32.4** | **93** | **27.0** | **109** | **51.9** | **12** | **2.4** | **57** | **47.5** | **176** | **85.9** |

**Table 7. Frequency and proportion of level of trust on the providers for COVID-19 vaccine by country and gender**

|  | Female | | Male | | Other | | Total | |
| --- | --- | --- | --- | --- | --- | --- | --- | --- |
|  | Freq. | % | Freq. | % | Freq. | % | Freq. | % |
| ***Overall*** |  |  |  |  |  |  |  |  |
| Not at all | 25 | 3.7 | 111 | 16.1 | 0 | 0.0 | 136 | 9.9 |
| A little | 217 | 31.8 | 162 | 23.5 | 0 | 0.0 | 379 | 27.6 |
| Moderately | 132 | 19.4 | 143 | 20.8 | 1 | 33.3 | 276 | 20.1 |
| Very much | 308 | 45.2 | 272 | 39.5 | 2 | 66.7 | 582 | 42.4 |
| Total | 682 | 100.0 | 688 | 100.0 | 3 | 100.0 | 1.373 | 100.0 |
| ***Ecuador*** |  |  |  |  |  |  |  |  |
| Not at all | 7 | 4.0 | 25 | 15.2 | 0 | 0.0 | 32 | 9.3 |
| A little | 36 | 20.3 | 37 | 22.6 | 0 | 0.0 | 73 | 21.2 |
| Moderately | 52 | 29.4 | 48 | 29.3 | 1 | 33.3 | 101 | 29.4 |
| Very much | 82 | 46.3 | 54 | 32.9 | 2 | 66.7 | 138 | 40.1 |
| Total | 177 | 100.0 | 164 | 100.0 | 3 | 100.0 | 344 | 100.0 |
| ***Nepal*** |  |  |  |  |  |  |  |  |
| Not at all | 1 | 1.1 | 1 | 0.8 | 0 | 0.0 | 2 | 1.0 |
| A little | 8 | 8.9 | 7 | 5.8 | 0 | 0.0 | 15 | 7.1 |
| Moderately | 10 | 11.1 | 23 | 19.2 | 0 | 0.0 | 33 | 15.7 |
| Very much | 71 | 78.9 | 89 | 74.2 | 0 | 0.0 | 160 | 76.2 |
| Total | 90 | 100.0 | 120 | 100.0 | 0 | 0.0 | 210 | 100.0 |
| ***Pakistan*** |  |  |  |  |  |  |  |  |
| Not at all | 14 | 5.4 | 76 | 31.7 | 0 | 0.0 | 90 | 18.0 |
| A little | 154 | 59.5 | 102 | 42.5 | 0 | 0.0 | 256 | 51.3 |
| Moderately | 47 | 18.1 | 43 | 17.9 | 0 | 0.0 | 90 | 18.0 |
| Very much | 44 | 17.0 | 19 | 7.9 | 0 | 0.0 | 63 | 12.6 |
| Total | 259 | 100.0 | 240 | 100.0 | 0 | 0.0 | 499 | 100.0 |
| ***Philippines*** |  |  |  |  |  |  |  |  |
| Not at all | 0 | 0.0 | 8 | 8.4 | 0 | 0.0 | 8 | 6.7 |
| A little | 4 | 16.0 | 12 | 12.6 | 0 | 0.0 | 16 | 13.3 |
| Moderately | 10 | 40.0 | 23 | 24.2 | 0 | 0.0 | 33 | 27.5 |
| Very much | 11 | 44.0 | 52 | 54.7 | 0 | 0.0 | 63 | 52.5 |
| Total | 25 | 100.0 | 95 | 100.0 | 0 | 0.0 | 120 | 100.0 |
| ***Rwanda*** |  |  |  |  |  |  |  |  |
| Not at all | 3 | 2.3 | 1 | 1.4 | 0 | 0.0 | 4 | 2.0 |
| A little | 15 | 11.4 | 4 | 5.8 | 0 | 0.0 | 19 | 9.5 |
| Moderately | 13 | 9.8 | 6 | 8.7 | 0 | 0.0 | 19 | 9.5 |
| Very much | 101 | 76.5 | 58 | 84.1 | 0 | 0.0 | 159 | 79.1 |
| Total | 132 | 100.0 | 69 | 100.0 | 0 | 0.0 | 201 | 100.0 |

**Table 8. Frequency and proportion of non-vaccination by reason and country**

|  | Overall  (n= 184) | | Ecuador (n=21) | | Nepal (n=8) | | Pakistan (n=151) | | Philippines (n=2) | | Rwanda (n=2) | |
| --- | --- | --- | --- | --- | --- | --- | --- | --- | --- | --- | --- | --- |
|  | Freq. | % | Freq. | % | Freq. | % | Freq. | % | Freq. | % | Freq. | % |
| I don't need the vaccine: rarely/never sick, I don't belong to the priority groups | 13 | 7.1 | 6 | 28.6 | 3 | 37.5 | 3 | 2.0 | 0 | 0.0 | 1 | 50.0 |
| Religious reasons | 3 | 1.6 | 0 | 0.0 | 2 | 25.0 | 0 | 0.0 | 0 | 0.0 | 1 | 50.0 |
| There is not much COVID-19 infection in the community | 7 | 3.8 | 0 | 0.0 | 3 | 37.5 | 4 | 2.6 | 0 | 0.0 | 0 | 0.0 |
| I do not need to protect myself | 9 | 4.9 | 2 | 9.5 | 2 | 25.0 | 5 | 3.3 | 0 | 0.0 | 0 | 0.0 |
| I do not need to protect my family/community | 2 | 1.1 | 0 | 0.0 | 0 | 0.0 | 2 | 1.3 | 0 | 0.0 | 0 | 0.0 |
| I am afraid of/do not like doctors/syringes | 11 | 6.0 | 4 | 19 | 1 | 12.5 | 6 | 4.0 | 0 | 0.0 | 0 | 0.0 |
| I want my body to develop natural immunity | 10 | 5.4 | 7 | 33.3 | 0 | 0.0 | 3 | 2.0 | 0 | 0.0 | 0 | 0.0 |
| I do not think the COVID-19 vaccines work | 18 | 9.8 | 7 | 33.3 | 0 | 0.0 | 11 | 7.3 | 0 | 0.0 | 0 | 0.0 |
| Risk of adverse reactions to COVID-19 vaccines | 61 | 33.2 | 6 | 28.6 | 1 | 12.5 | 54 | 35.8 | 0 | 0.0 | 0 | 0.0 |
| Adverse reactions to previous vaccines (others than the COVID-19 vaccine) | 7 | 3.8 | 2 | 9.05 | 0 | 0.0 | 5 | 3.3 | 0 | 0.0 | 0 | 0.0 |
| There is too little experience with the use of the vaccine | 8 | 4.3 | 2 | 9.5 | 0 | 0.0 | 6 | 4.0 | 0 | 0.0 | 0 | 0.0 |
| I am not comfortable with the new COVID-19 vaccine | 10 | 5.4 | 1 | 4.8 | 2 | 25.0 | 7 | 4.6 | 0 | 0.0 | 0 | 0.0 |
| I do not trust the recommendation from the health authorities/municipality | 8 | 4.3 | 5 | 9.5 | 0 | 0.0 | 3 | 2.0 | 0 | 0.0 | 0 | 0.0 |
| Information from the media | 1 | 0.8 | 0 | 0.0 | 0 | 0.0 | 1 | 0.7 | 0 | 0.0 | 0 | 0.0 |
| Political reasons (antigovernment feelings) | 1 | 0.5 | 0 | 0.0 | 0 | 0.0 | 1 | 0.7 | 0 | 0.0 | 0 | 0.0 |

**Table 9. Type of difficulties to access COVID-19 vaccine by country**

|  | Overall  (n=1378) | | Ecuador (n=344) | | Nepal (n=210) | | Pakistan  (n=499) | | Philippines (n=120) | | Rwanda (n=205) | |
| --- | --- | --- | --- | --- | --- | --- | --- | --- | --- | --- | --- | --- |
|  | Freq. | % | Freq. | % | Freq. | % | Freq. | % | Freq. | % | Freq. | % |
| Nothing. It is not difficult | 751 | 54.5 | 166 | 48.3 | 124 | 59.0 | 237 | 47.5 | 28 | 23.3 | 196 | 95.6 |
| The COVID-19 vaccine is not available for a given group | 66 | 4.8 | 7 | 2.0 | 3 | 1.4 | 48 | 9.6 | 8 | 6.7 | 0 | 0.0 |
| Vaccination costs too much | 27 | 2.0 | 1 | 0.3 | 0 | 0.0 | 24 | 4.8 | 2 | 1.7 | 0 | 0.0 |
| They can't go on their own (they have physical limitation) | 79 | 5.7 | 5 | 1.5 | 7 | 3.3 | 57 | 11.4 | 10 | 8.3 | 0 | 0.0 |
| The vaccination site is too far away | 99 | 7.2 | 17 | 4.9 | 7 | 3.3 | 61 | 12.2 | 14 | 11.7 | 0 | 0.0 |
| Opening hours are inconvenient | 37 | 2.7 | 6 | 1.7 | 3 | 1.4 | 24 | 4.8 | 4 | 3.3 | 0 | 0.0 |
| People are turned away without vaccination | 50 | 3.6 | 17 | 4.9 | 4 | 1.9 | 27 | 5.4 | 2 | 1.7 | 0 | 0.0 |
| The waiting time is too long | 114 | 8.3 | 45 | 13.1 | 9 | 4.3 | 31 | 6.2 | 25 | 20.8 | 4 | 1.9 |
| It is difficult to register for vaccination | 57 | 4.1 | 3 | 0.9 | 1 | 0.5 | 46 | 9.2 | 7 | 4.2 | 0 | 0.0 |
| They do not have the internet or a smartphone to register for vaccination | 30 | 2.2 | 0 | 0.0 | 1 | 0.5 | 29 | 5.8 | 0 | 0.0 | 0 | 0.0 |

**Graph 2: Witness of any kind of discrimination by country**

**
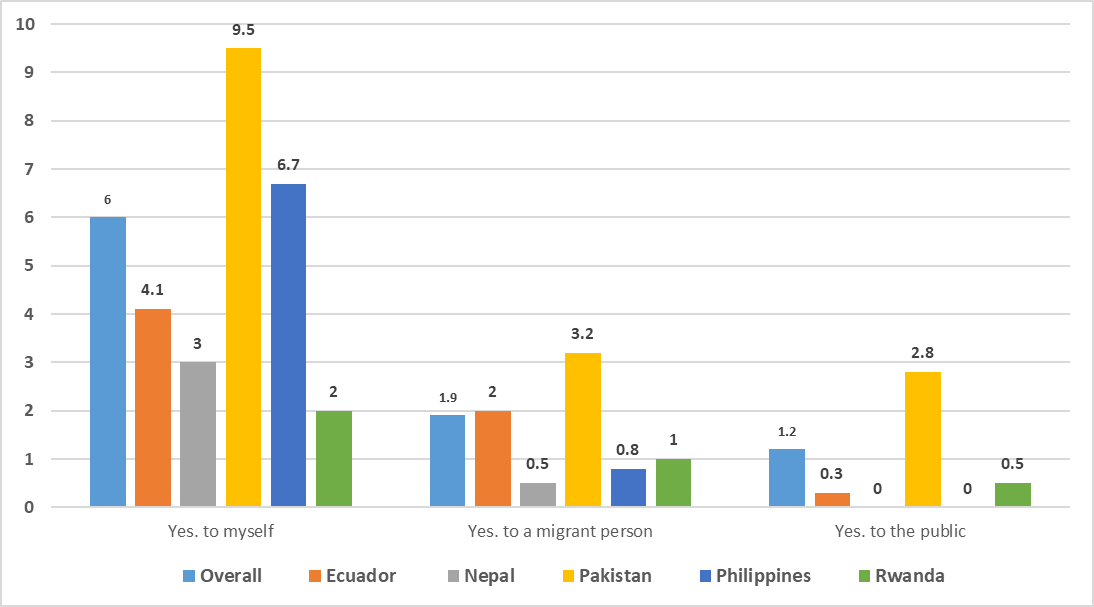
**

**Table 10. Distribution of the kinds of discrimination witnessed by country and gender**

|  | Female | | Male | | Other | | Total | |
| --- | --- | --- | --- | --- | --- | --- | --- | --- |
|  | Freq. | % | Freq. | Freq. | Freq. | Freq. | Freq. | % |
| ***Overall*** |  |  |  |  |  |  |  |  |
| Yes, to myself | 25 | 3.7 | 61 | 8.8 | 0 | 0.0 | 86 | 6.2 |
| Yes, to a migrant person | 10 | 1.5 | 16 | 2.3 | 0 | 0.0 | 26 | 1.9 |
| Yes, to the public | 7 | 1.0 | 7 | 1.0 | 0 | 0.0 | 14 | 1.0 |
| I do not witness any act of discrimination | 642 | 93.9 | 607 | 87.8 | 3 | 100.0 | 1252 | 90.9 |
| Total | 684 | 100.0 | 691 | 100.0 | 3 | 100.0 | 1378 | 100.0 |
| ***Ecuador*** |  |  |  |  |  |  |  |  |
| Yes, to myself | 8 | 4.5 | 6 | 3.7 | 0 | 0.0 | 14 | 4.1 |
| Yes, to a migrant person | 6 | 3.4 | 1 | 0.6 | 0 | 0.0 | 7 | 2.0 |
| Yes, to the public | 0 | 0.0 | 1 | 0.6 | 0 | 0.0 | 1 | 0.3 |
| I do not witness any act of discrimination | 163 | 92.1 | 156 | 95.1 | 3 | 100.0 | 322 | 93.6 |
| Total | 177 | 100.0 | 164 | 100.0 | 3 | 100.0 | 344 | 100.0 |
| ***Nepal*** |  |  |  |  |  |  |  |  |
| Yes, to myself | 3 | 3.5 | 3 | 2.6 | 0 | 0.0 | 6 | 3.0 |
| Yes, to a migrant person | 0 | 0.0 | 1 | 0.9 | 0 | 0.0 | 1 | 0.5 |
| Yes, to the public | 0 | 0.0 | 0 | 0.0 | 0 | 0.0 | 0 | 0.0 |
| I do not witness any act of discrimination | 83 | 96.5 | 112 | 96.6 | 0 | 0.0 | 195 | 96.5 |
| Total | 86 | 100.0 | 116 | 100.0 | 0 | 0.0 | 202 | 100.0 |
| ***Pakistan*** |  |  |  |  |  |  |  |  |
| Yes, to myself | 7 | 2.7 | 46 | 19.2 | 0 | 0.0 | 53 | 10.6 |
| Yes, to a migrant person | 3 | 1.2 | 14 | 5.8 | 0 | 0.0 | 17 | 3.4 |
| Yes, to the public | 7 | 2.7 | 6 | 2.5 | 0 | 0.0 | 13 | 2.6 |
| I do not witness any act of discrimination | 242 | 93.4 | 174 | 72.5 | 0 | 0.0 | 416 | 83.4 |
| Total | 259 | 100.0 | 240 | 100.0 | 0 | 0.0 | 499 | 100.0 |
| ***Philippines*** |  |  |  |  |  |  |  |  |
| Yes, to myself | 2 | 8.0 | 6 | 6.3 | 0 | 0.0 | 8 | 7.5 |
| Yes, to a migrant person | 1 | 4.0 | 0 | 0.0 | 0 | 0.0 | 1 | 0.0 |
| Yes, to the public | 0 | 0.0 | 0 | 0.0 | 0 | 0.0 | 0 | 0.0 |
| I do not witness any act of discrimination | 22 | 88.0 | 89 | 93.7 | 0 | 0.0 | 111 | 92.5 |
| Total | 25 | 100.0 | 95 | 100.0 | 0 | 0.0 | 120 | 100.0 |
| ***Rwanda*** |  |  |  |  |  |  |  |  |
| Yes, to myself | 4 | 3.0 | 0 | 0.0 | 0 | 0.0 | 4 | 2.0 |
| Yes, to a migrant person | 2 | 1.5 | 0 | 0.0 | 0 | 0.0 | 2 | 1.0 |
| Yes, to the public | 1 | 0.8 | 0 | 0.0 | 0 | 0.0 | 1 | 0.5 |
| I do not witness any act of discrimination | 128 | 96.2 | 72 | 100.0 | 0 | 0.0 | 200 | 97.6 |
| Total | 133 | 100.0 | 72 | 100.0 | 0 | 0.0 | 205 | 100.0 |

**Table 11. Sources of discrimination witnessed by country**

|  | Overall (n=126) | | Ecuador (n=22) | | Nepal (n=7) | | Pakistan (n=83) | | Philippines (n=9) | | Rwanda (n=5) | |
| --- | --- | --- | --- | --- | --- | --- | --- | --- | --- | --- | --- | --- |
|  | Freq. | % | Freq. | % | Freq. | % | Freq. | % | Freq. | % | Freq. | % |
| Security personnel | 39 | 31.0 | 6 | 27.3 | 1 | 14.3 | 28 | 33.7 | 2 | 22.2 | 2 | 40.0 |
| Health center staff | 72 | 57.1 | 13 | 59.1 | 2 | 28.6 | 50 | 60.2 | 4 | 44.4 | 3 | 60.0 |
| Local population | 16 | 12.7 | 3 | 13.6 | 1 | 14.3 | 8 | 9.6 | 3 | 33.3 | 1 | 20.0 |

**Table 12. Frequency and proportion of those who perceive any differences in the way you were treated for vaccinations compared to the majority population by country, gender, and migration status**

|  | Overall (n=1194) | | Ecuador (n=323) | | Nepal (n=202) | | Pakistan (n=348) | | Philippines (n=118) | | Rwanda (n=204) | |
| --- | --- | --- | --- | --- | --- | --- | --- | --- | --- | --- | --- | --- |
|  | Freq. | % | Freq. | % | Freq. | % | Freq. | % | Freq. | % | Freq. | % |
| ***Gender*** |  |  |  |  |  |  |  |  |  |  |  |  |
| Female | 86 | 15.0 | 11 | 6.4 | 2 | 2.3 | 61 | 38.1 | 2 | 8.0 | 10 | 7.6 |
| Male | 98 | 15.9 | 6 | 4.0 | 4 | 3.4 | 81 | 43.1 | 4 | 4.3 | 3 | 4.2 |
| Other | 1 | 33.3 | 1 | 33.3 | 0 | 0.0 | 0 | 0.0 | 0 | 0.0 | 0 | 0.0 |
| ***Migration status*** |  |  |  |  |  |  |  |  |  |  |  |  |
| Regular | 104 | 21.2 | 5 | 5.7 | 4 | 6.6 | 90 | 37.7 | 3 | 5.4 | 2 | 4.3 |
| Irregular | 53 | 13.9 | 11 | 5.0 | 0 | 0.0 | 41 | 50.0 | 1 | 3.0 | 0 | 0.0 |
| Refugee | 28 | 8.7 | 2 | 13.3 | 2 | 2.0 | 11 | 40.7 | 2 | 6.9 | 11 | 7.2 |
| **Total** | **185** | **15.5** | **18** | **5.6** | **6** | **3.0** | **142** | **40.8** | **6** | **5.1** | **13** | **6.4** |

**Table 13. Frequency and proportion of being denied access to vaccination due to lack of having an identity document, by country, gender, and migration status**

|  | Overall (n=1378) | | Ecuador (n=344) | | Nepal (n=210) | | Pakistan (n=499) | | Philippines (n=120) | | Rwanda (n=205) | |
| --- | --- | --- | --- | --- | --- | --- | --- | --- | --- | --- | --- | --- |
|  | Freq. | % | Freq. | % | Freq. | % | Freq. | % | Freq. | % | Freq. | % |
| ***Gender*** |  |  |  |  |  |  |  |  |  |  |  |  |
| Female | 30 | 4.4 | 11 | 6.2 | 2 | 2.2 | 3 | 1.2 | 2 | 8.0 | 12 | 9.0 |
| Male | 55 | 8.0 | 14 | 8.5 | 6 | 5.0 | 15 | 6.3 | 9 | 9.5 | 11 | 15.3 |
| Other | 0 | 0.0 | 0 | 0.0 | 0 | 0.0 | 0 | 0.0 | 0 | 0.0 | 0 | 0.0 |
| ***Migration status*** |  |  |  |  |  |  |  |  |  |  |  |  |
| Regular | 27 | 4.5 | 6 | 6.7 | 3 | 4.5 | 9 | 2.6 | 5 | 8.9 | 4 | 8.5 |
| Irregular | 32 | 7.4 | 17 | 7.2 | 2 | 4.7 | 7 | 6.2 | 4 | 12.1 | 2 | 50.0 |
| Refugee | 26 | 7.5 | 2 | 11.8 | 3 | 3.0 | 2 | 4.8 | 2 | 6.5 | 17 | 11.0 |
| **Total** | **85** | **6.2** | **25** | **7.3** | **8** | **3.8** | **18** | **3.6** | **11** | **9.2** | **23** | **11.2** |

Table 14. Frequency and proportion of main sources of information on COVID-19 vaccination by country

|  | Overall  (n=1378) | | Ecuador  (n=344) | | | Nepal  (n=210) | | | Pakistan  (n=499) | | | Philippines (n=120) | | Rwanda (n=205) | |
| --- | --- | --- | --- | --- | --- | --- | --- | --- | --- | --- | --- | --- | --- | --- | --- |
|  | Freq. | % | Freq. | % | Freq. | | % | Freq. | | % | Freq. | | % | Freq. | % |
| Local newspapers. radio and/or television channels | 687 | 49.9 | 153 | 44.5 | 67 | | 31.9 | 310 | | 62.1 | 30 | | 25.0 | 127 | 61.7 |
| Foreign newspapers. radio and/or television channels | 282 | 20.5 | 44 | 12.8 | 41 | | 19.5 | 152 | | 30.5 | 20 | | 16.7 | 25 | 12.1 |
| Local authorities’ statements | 248 | 18 | 64 | 18.6 | 34 | | 16.2 | 74 | | 14.8 | 17 | | 14.2 | 59 | 28.6 |
| Local government websites | 153 | 11.1 | 77 | 22.4 | 2 | | 1.0 | 38 | | 7.6 | 17 | | 14.2 | 19 | 9.2 |
| Brochures | 131 | 9.5 | 42 | 12.2 | 1 | | 0.5 | 41 | | 8.2 | 0 | | 0.0 | 47 | 23.3 |
| Social media | 402 | 29.2 | 157 | 45.6 | 49 | | 23.3 | 82 | | 16.4 | 61 | | 50.8 | 53 | 25.7 |
| Family and/or friends in your home country | 294 | 21.3 | 103 | 29.9 | 47 | | 22.4 | 119 | | 23.8 | 7 | | 5.8 | 18 | 8.7 |
| Family and/or friends in the country you are currently living | 298 | 21.6 | 110 | 32.0 | 41 | | 19.5 | 82 | | 16.4 | 20 | | 16.7 | 45 | 21.8 |
| Camp staff | 295 | 21.4 | 23 | 6.7 | 72 | | 34.3 | 71 | | 14.2 | 0 | | 0.0 | 129 | 62.9 |
| At your workplace | 147 | 10.7 | 48 | 14.0 | 11 | | 5.2 | 61 | | 12.2 | 10 | | 8.3 | 17 | 8.3 |
| Internet and/or social networks | 312 | 22.6 | 192 | 55.8 | 12 | | 5.7 | 18 | | 3.6 | 50 | | 41.7 | 40 | 19.4 |
| NGO’s | 152 | 11 | 73 | 21.2 | 19 | | 9.0 | 18 | | 3.6 | 3 | | 2.5 | 39 | 18.9 |
| I have not received information about COVID-19 vaccination | 7 | 0.5 | 1 | 0.3 | 0 | | 0.0 | 6 | | 1.2 | 0 | | 0.0 | 0 | 0.0 |

**Table 15. Frequency and proportion of those who think that the national government's campaign to raise awareness of the benefits of the vaccine has been successful in getting migrants to go to vaccination centres by country, gender, and migration status, age, duration in the host country, and level of education**

|  | Overall (n=1378) | | Ecuador (n=344) | | Nepal (n=210) | | Pakistan (n=499) | | Philippines (n=120) | | Rwanda (n=205) | |
| --- | --- | --- | --- | --- | --- | --- | --- | --- | --- | --- | --- | --- |
|  | Freq. | % | Freq. | % | Freq. | % | Freq. | % | Freq. | % | Freq. | % |
| ***Gender*** |  |  |  |  |  |  |  |  |  |  |  |  |
| Female | 438 | 64.0 | 141 | 79.7 | 57 | 63.3 | 120 | 46.3 | 16 | 64.0 | 104 | 78.2 |
| Male | 444 | 64.3 | 114 | 69.5 | 85 | 70.8 | 106 | 44.2 | 76 | 80.0 | 63 | 87.5 |
| Other | 2 | 66.7 | 2 | 66.7 | 0 | 0.0 | 0 | 0.0 | 0 | 0.0 | 0 | 0.0 |
| ***Migration status*** |  |  |  |  |  |  |  |  |  |  |  |  |
| Regular | 343 | 56.9 | 70 | 77.8 | 40 | 60.6 | 155 | 45.1 | 41 | 73.2 | 37 | 78.7 |
| Irregular | 297 | 69.1 | 178 | 75.1 | 29 | 67.4 | 58 | 51.3 | 28 | 84.8 | 4 | 100.0 |
| Refugee | 244 | 70.7 | 9 | 52.9 | 73 | 72.3 | 13 | 31.0 | 23 | 74.2 | 126 | 81.8 |
| ***Age group*** |  |  |  |  |  |  |  |  |  |  |  |  |
| 18 – 29 | 236 | 66.9 | 77 | 67.5 | 35 | 76.1 | 39 | 46.4 | 65 | 76.5 | 20 | 80.0 |
| 30 – 49 | 355 | 70.7 | 133 | 78.7 | 44 | 67.7 | 52 | 44.8 | 22 | 75.9 | 104 | 83.9 |
| 50 – 64 | 170 | 59.0 | 41 | 78.8 | 34 | 65.4 | 61 | 43.3 | 4 | 80.0 | 30 | 78.9 |
| 65 or more | 123 | 52.8 | 6 | 66.7 | 29 | 61.7 | 74 | 46.8 | 1 | 100.0 | 13 | 72.2 |
| ***Duration in the host country*** |  |  |  |  |  |  |  |  |  |  |  |  |
| < 1 year | 78 | 66.7 | 57 | 66.3 | 7 | 63.6 | 12 | 70.6 | 1 | 50.0 | 1 | 100.0 |
| 1 – 5 years | 257 | 78.4 | 163 | 78.4 | 11 | 78.6 | 4 | 57.1 | 53 | 75.7 | 26 | 89.7 |
| More than 5 years | 549 | 58.8 | 37 | 74.0 | 124 | 67.0 | 210 | 44.2 | 38 | 79.2 | 140 | 80.9 |
| ***Education*** |  |  |  |  |  |  |  |  |  |  |  |  |
| Formal education not completed | 217 | 50.9 | 4 | 57.1 | 15 | 60.0 | 167 | 46.4 | 0 | 0.0 | 31 | 91.2 |
| Primary education | 94 | 61.8 | 17 | 73.9 | 26 | 61.9 | 28 | 48.3 | 0 | 0.0 | 23 | 79.3 |
| Secondary education completed | 166 | 70.6 | 93 | 73.2 | 19 | 65.5 | 12 | 41.4 | 28 | 80.0 | 14 | 93.8 |
| Secondary education not completed | 81 | 68.6 | 28 | 68.3 | 23 | 76.7 | 7 | 36.8 | 2 | 100.0 | 21 | 80.8 |
| Technical education | 18 | 78.3 | 15 | 83.3 | 0 | 0.0 | 0 | 0.0 | 1 | 100.0 | 2 | 50.0 |
| Bachelor degree | 156 | 79.2 | 75 | 80.6 | 8 | 100.0 | 1 | 20.0 | 54 | 79.4 | 18 | 78.3 |
| Postgraduate degree | 23 | 65.7 | 5 | 62.5 | 1 | 100.0 | 0 | 0.0 | 7 | 50.0 | 10 | 83.3 |
| None of the above | 105 | 68.2 | 10 | 83.3 | 50 | 66.7 | 8 | 47.1 | 0 | 0.0 | 37 | 74.0 |
| I prefer not to answer | 24 | 63.2 | 10 | 66.7 | 0 | 0.0 | 3 | 27.3 | 0 | 0.0 | 11 | 91.7 |
| **Total** | **884** | **64.2** | **257** | **74.7** | **142** | **67.6** | **226** | **45.3** | **14** | **11.7** | **167** | **81.5** |

**Table 16. Frequency and proportion of those who consider that the health centres set up at the national level are accessible in their community by country, gender, migration status, age, duration in the host country and level of education**

|  | Overall  (n= 1378) | | Ecuador  (n=344) | | Nepal (n=210) | | Pakistan (n=499) | | Philippines (n=120) | | Rwanda (n=205) | |
| --- | --- | --- | --- | --- | --- | --- | --- | --- | --- | --- | --- | --- |
|  | Freq. | % | Freq. | % | Freq. | % | Freq. | % | Freq. | % | Freq. | % |
| ***Gender*** |  |  |  |  |  |  |  |  |  |  |  |  |
| Female | 472 | 69.0 | 155 | 87.6 | 64 | 71.1 | 112 | 43.2 | 20 | 80.0 | 121 | 91.0 |
| Male | 476 | 68.9 | 136 | 82.9 | 82 | 68.3 | 109 | 45.4 | 83 | 87.4 | 66 | 91.7 |
| Other | 2 | 66.7 | 2 | 66.7 | 0 | 0.0 | 0 | 0.0 | 0 | 0.0 | 0 | 0.0 |
| ***Migration status*** |  |  |  |  |  |  |  |  |  |  |  |  |
| Regular | 345 | 57.2 | 74 | 82.2 | 42 | 63.6 | 141 | 41.0 | 49 | 87.5 | 39 | 83.0 |
| Irregular | 338 | 78.6 | 208 | 87.8 | 33 | 76.7 | 66 | 58.4 | 27 | 81.8 | 4 | 100.0 |
| Refugee | 267 | 77.4 | 11 | 64.7 | 71 | 70.3 | 14 | 33.3 | 27 | 87.1 | 144 | 93.5 |
| ***Age group*** |  |  |  |  |  |  |  |  |  |  |  |  |
| 18 – 29 | 262 | 74.0 | 92 | 80.7 | 33 | 71.7 | 39 | 46.4 | 75 | 88.2 | 23 | 92.0 |
| 30 – 49 | 375 | 74.6 | 145 | 85.8 | 45 | 69.2 | 51 | 44.0 | 22 | 75.9 | 112 | 90.3 |
| 50 – 64 | 189 | 65.6 | 48 | 92.3 | 38 | 73.1 | 62 | 44.0 | 5 | 100.0 | 36 | 94.7 |
| 65 or more | 124 | 53.2 | 8 | 88.9 | 30 | 63.8 | 69 | 43.7 | 1 | 100.0 | 16 | 88.9 |
| ***Duration in the host country*** |  |  |  |  |  |  |  |  |  |  |  |  |
| < 1 year | 96 | 82.1 | 73 | 84.9 | 8 | 72.7 | 13 | 76.5 | 1 | 50.0 | 1 | 100.0 |
| 1 – 5 years | 276 | 84.1 | 179 | 86.1 | 10 | 71.4 | 3 | 42.9 | 60 | 85.7 | 24 | 82.2 |
| More than 5 years | 578 | 62.0 | 41 | 82.0 | 128 | 69.2 | 205 | 43.2 | 42 | 87.5 | 162 | 92.6 |
| ***Education*** |  |  |  |  |  |  |  |  |  |  |  |  |
| Formal education not completed | 214 | 50.2 | 4 | 57.1 | 16 | 64.0 | 160 | 44.4 | 0 | 0.0 | 34 | 100.0 |
| Primary education | 100 | 65.8 | 20 | 87.0 | 25 | 59.5 | 29 | 50.0 | 0 | 0.0 | 26 | 89.7 |
| Secondary education completed | 187 | 79.6 | 108 | 85.0 | 16 | 55.2 | 16 | 55.2 | 32 | 91.4 | 15 | 100.0 |
| Secondary education not completed | 92 | 78.0 | 35 | 85.4 | 23 | 76.7 | 7 | 36.8 | 2 | 100.0 | 25 | 96.2 |
| Technical education | 20 | 87.0 | 16 | 88.9 | 0 | 0.0 | 0 | 0.0 | 0 | 0.0 | 4 | 100.0 |
| Bachelor degree | 163 | 82.7 | 79 | 84.9 | 6 | 75.0 | 1 | 20.0 | 58 | 85.3 | 19 | 82.6 |
| Postgraduate degree | 28 | 80.0 | 7 | 87.5 | 1 | 100.0 | 0 | 0.0 | 11 | 78.6 | 9 | 75.0 |
| None of the above | 118 | 76.6 | 11 | 91.7 | 59 | 78.7 | 5 | 29.4 | 0 | 0.0 | 43 | 86.0 |
| I prefer not to answer | 28 | 73.7 | 13 | 86.7 | 0 | 0.0 | 3 | 27.3 | 0 | 0.0 | 12 | 100.0 |
| **Total** | **950** | **68.9** | **293** | **85.2** | **146** | **69.5** | **221** | **44.3** | **103** | **85.8** | **187** | **91.3** |

**Graph 3. Facilitators identified by country**


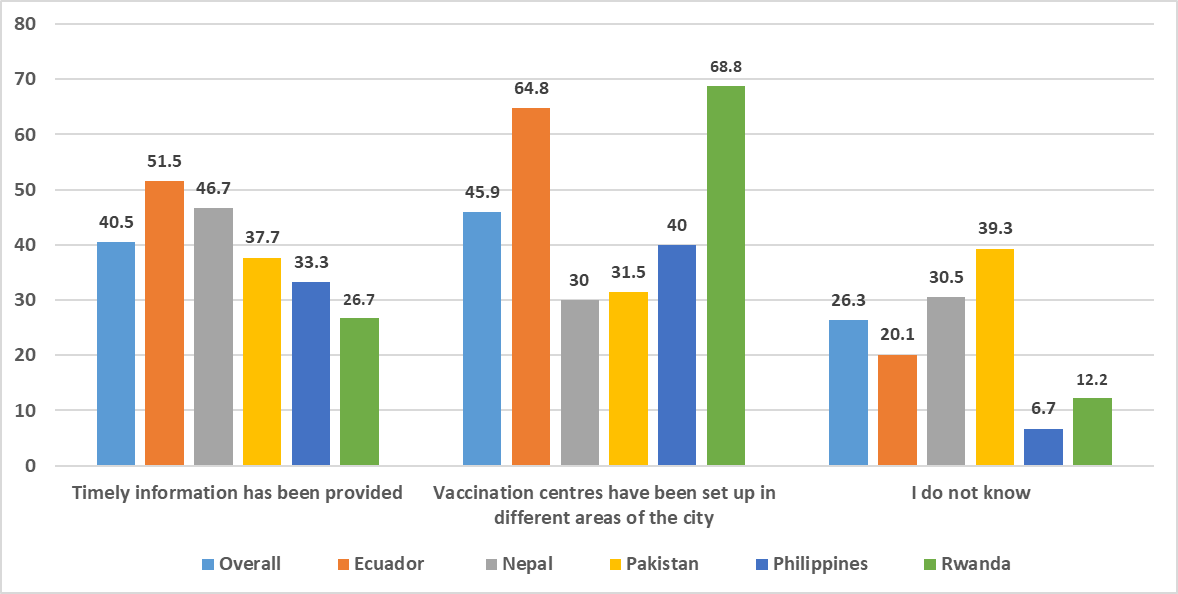


**Table 17. Frequency and proportion of the type of payment by country and migration status**

|  | Overall  (n=1194) | | Ecuador (n=323) | | Nepal  (n=202) | | Pakistan (n=348) | | Philippines (n=118) | | Rwanda (n=203) | |
| --- | --- | --- | --- | --- | --- | --- | --- | --- | --- | --- | --- | --- |
|  | Freq. | % | Freq. | % | Freq. | % | Freq. | % | Freq. | % | Freq. | % |
| Yes, I paid to get an appointment | 22 | 1.8 | 2 | 0.6 | 0 | 0.0 | 19 | 5.5 | 0 | 0.0 | 1 | 0.5 |
| Yes, I bought the syringes | 1 | 0.1 | 0 | 0.0 | 0 | 0.0 | 1 | 0.3 | 0 | 0.0 | 0 | 0.0 |
| Yes, I paid to get a vaccination certificate | 8 | 0.7 | 0 | 0.0 | 0 | 0.0 | 8 | 2.3 | 0 | 0.0 | 0 | 0.0 |
| No, it was totally free of charge | 1109 | 92.9 | 319 | 98.8 | 202 | 100.0 | 273 | 78.4 | 113 | 94.2 | 202 | 99.5 |

**Table 18. Reasons of vaccination by country and migration status**

|  | Overall  (n=1194) | | Ecuador (n=323) | | Nepal (n=202) | | Pakistan  (n=348) | | Philippines (n=118) | | Rwanda (n=203) | |
| --- | --- | --- | --- | --- | --- | --- | --- | --- | --- | --- | --- | --- |
|  | Freq. | % | Freq. | % | Freq. | % | Freq. | % | Freq. | % | Freq. | % |
| The vaccine is highly reliable | 683 | 57.2 | 195 | 60.4 | 184 | 91.1 | 34 | 9.8 | 95 | 80.5 | 175 | 86.2 |
| The vaccine is a government requirement | 696 | 58.3 | 233 | 72.1 | 174 | 86.1 | 30 | 8.6 | 101 | 85.6 | 158 | 77.8 |
| The vaccine protects my family. friends. and community | 741 | 62.1 | 241 | 74.6 | 190 | 94.1 | 27 | 7.8 | 97 | 82.2 | 186 | 91.6 |
| The vaccine is a requirement to enter establishments | 709 | 59.4 | 254 | 78.6 | 168 | 83.2 | 24 | 6.9 | 102 | 86.4 | 161 | 79.3 |
| Getting vaccinated is my duty | 725 | 60.7 | 259 | 80.2 | 168 | 83.2 | 19 | 5.5 | 94 | 79.7 | 185 | 91.1 |
| I get vaccinated because it's safe | 670 | 56.1 | 207 | 64.1 | 161 | 79.7 | 31 | 8.9 | 85 | 72.0 | 186 | 91.6 |
| I would not like to get vaccinated, but I am forced | 174 | 14.6 | 111 | 34.4 | 2 | 1.0 | 18 | 5.2 | 29 | 24.6 | 14 | 6.9 |
