# Supplementary material for: Deployment and uptake of COVID-19 vaccines for refugees and migrants in regular and irregular situations: a mixed-method multicountry study
Source: BMJ Open. 2025 Feb 2;15(1):e087629. doi: 10.1136/bmjopen-2024-087629 (PMC11904359; doi:10.1136/bmjopen-2024-087629)
Supplement: online supplemental file 2 [file bmjopen-15-1-s002.docx]

**Focus Group Interview guide**

1. **Welcome**
2. **Introduction of the objective of the interview**

My name is _____, my assistant’s name is _____and I would like to thank you for meeting us today for this interview on the status of COVID-19 vaccine access and uptake, and challenges and enablers in accessing COVID-19 vaccines by refugees and migrants.

1. **Introductions**

Before we start, I’d like to go around the room and have everyone introduce themselves.

1. **Oral consent**

*Before we begin with this interview, I would like to go over the informed consent. Read the script for informed consent to the participant after the initial introduction of the objective of the interview. If you have any questions regarding the interview, I will be happy to answer them.*

1. **Explaining the rules**

There are a few rules to keep in mind while participating today:

a) We expect everyone to be an active participant.

b) There are no “right” or “wrong” answers.

c) It is important not to interrupt others while they are talking.

d) The interview will be recorder. Note taking will be used only for analysis.

f) All you talk about today will remain anonymous.

**Provide a brief situational update**

*Describe briefly the current circumstances surrounding COVID-19 pandemic in the country/community? and state of the vaccination process.*

**The main interview**

*Use the questions below. Adapt questions and add more probes as needed to elicit detailed information.*

**Introduction**

1. Which are the main refugee and migrant groups in your community/country?

2.In most countries, the COVID-19 pandemic has had numerous impacts, including creating historic morbidity and mortality, overburdened health systems, education loss for children, and loss of livelihoods for workers. Are there any specific consequences of COVID-19 pandemic in your community/country?

**COVID-19 Vaccination: Leadership and governance**

1.Could you describe how local COVID-19 vaccine policies are inclusive or not of refugee and migrant populations? Why? Can you provide examples of migrant-inclusive or migrant-exclusive vaccine policies? Can all migrants access COVID-19 vaccines?

2.Could you tell us whether refugees’ and migrants’ access to the COVID-19 vaccine is equal across the country?

**COVID-19 Vaccination: financing**

1.Could you tell us whether COVID-19 vaccines are free for refugees and migrants? Are there any migrant groups that are expected to cover the cost of the vaccine?

**COVID-19 Vaccination: health information systems**

1.Do you know about any COVID-19 vaccination campaigns for refuges and migrants in your community/country? Can you provide some examples? Who lunched the campaigns?

2.Could you tell us if all refugees and migrant groups have access to the information on COVID-19 vaccines?

**COVID-19 Vaccination: service delivery and access to essential medicines**

1.What, in your opinion, affects the willingness of refugees and migrants to seek vaccination?

2.What are the barriers to refugees’ and migrants’ access to and uptake of COVID-19 vaccines in your community/country?

probe on:

-vaccination policies

-language

-disinformation/misinformation (e.g., conspiracy theories or rumours)

-attitudes toward vaccine

-fear of side effects

-trust in healthcare system

-fear of sharing personal data collected at vaccination distribution sites with public health workers

-distance to vaccination distribution sites

-vaccination fee

-digital competency to register

-fear of deportation or jeopardising (legal) status

3.What facilitates refugees’ and migrants’ access to and uptake of COVID-19 vaccines in your community/country?

probe on:

-vaccination policies

-public health information campaigns (e.g., by WHO, national health ministry, civil society organisations, community and faith organisations)

-information available in migrants’ languages

-experience of the death of relatives or friends

-personal health status or comorbidity

-facilitating access to community services (e.g., education)

-facilitating easier movement within community/country

-obliged to receive the vaccine (i.e. job requirements)

4. Could you mention some important differences in access to COVID-19 vaccine among the different migrant groups (refugees, migrants in irregular situation, and migrants in regular situation)?

**Closing the interview**

1.We talked about the barriers for refugees and migrants to access the COVID-19 vaccines. What would be your top three suggestions/recommendations/actions on how to overcome the specific barriers you mentioned?

2.Is there anything else you would like to share with us?

I would like to thank you for your time and for sharing your thoughts and experiences with me. It will help us to understand better the status of COVID-19 vaccine access and uptake in refugee and migrant populations.
