# Supplementary material for: Deployment and uptake of COVID-19 vaccines for refugees and migrants in regular and irregular situations: a mixed-method multicountry study
Source: BMJ Open. 2025 Feb 2;15(1):e087629. doi: 10.1136/bmjopen-2024-087629 (PMC11904359; doi:10.1136/bmjopen-2024-087629)
Supplement: online supplemental file 3 [file bmjopen-15-1-s003.docx]

**A semi-structured guide for Key Informant Interviews**

1. **Introduction of the objective of the interview**

My name is _____, and I would like to thank you for meeting me today for this interview on the status of COVID-19 vaccine access and uptake, and challenges and enablers in accessing COVID-19 vaccines by refugees and migrants.

1. **Oral consent**

Before we begin with this interview, I would like to go over the informed consent. *Read the script for informed consent to the participant after the initial introduction of the objective of the intervi*ew. If you have any questions regarding the interview, I will be happy to answer them.

1. **Provide a brief situational update for the KII (Key Informant Interview).**

*Describe briefly the current circumstances surrounding COVID-19 pandemic in the country/community? and state of the vaccination process.*

# The main interview

*Use the questions below. Adapt questions and add more probes as needed to elicit detailed information.*

## Introduction

1. Which are the main refugee and migrant groups in your community/country?

2.In most countries, the COVID-19 pandemic has had numerous impacts, including creating historic morbidity and mortality, overburdened health systems, education loss for children, and loss of livelihoods for workers. Are there any specific consequences of COVID-19 pandemic in your community/country?

## COVID-19 Vaccination: Leadership and governance

1.Could you describe how national or local COVID-19 vaccine policies are or are not inclusive of refugee and migrant populations? Why? Can you provide examples of migrant-inclusive or migrant-exclusive vaccine policies? Can all migrants access COVID-19 vaccines?

2.Could you tell us whether refugees’ and migrants’ access to the COVID-19 vaccine is equal across different regions of the country?

## COVID-19 Vaccination: financing

1.Could you tell us whether COVID-19 vaccines are free for refugees and migrants? Are there any migrant groups that are expected to cover the cost of the vaccine?

## COVID-19 Vaccination: health information systems

1.Do you know about any COVID-19 vaccination campaigns for refugees and migrants in your community/country? Can you provide some examples? Who launched the campaigns?

## COVID-19 Vaccination: service delivery and access to essential medicines

1. What, in your opinion, affects the willingness of refugees and migrants to seek vaccination?

2.What are the barriers to refugees’ and migrants’ access to COVID-19 vaccines in your community/country?

probe on:

-vaccination policies

-language

-disinformation/misinformation (e.g., conspiracy theories or rumours)

-attitudes toward vaccine

-fear of side effects

-trust in healthcare system

-fear of sharing personal data collected at vaccination distribution sites with public health workers

-distance to vaccination distribution sites

-vaccination fee

-digital competency to register

-fear of deportation or jeopardising (legal) status

3.What facilitates refugees’ and migrants’ access to f COVID-19 vaccines in your community/country?

probe on:

-vaccination policies

-public health information campaigns (e.g., by WHO, national health ministry, civil society organisations, community and faith organisations)

-information available in migrants’ languages

-experience of the death of relatives or friends

-personal health status or comorbidity

-facilitating access to community services (e.g., education)

-facilitating easier movement within community/country

-obliged to receive the vaccine (i.e. job requirements)

4. Could you mention some important differences in access to COVID-19 vaccine among the different migrant groups (refugees, migrants in irregular situation, and migrants in regular situation)?

## Closing the interview

1.We talked about the barriers for refugees and migrants to access the COVID-19 vaccines. What would be your top three suggestions/recommendations/actions on how to overcome the specific barriers you mentioned?

2.Is there anything else you would like to share with me?

I would like to thank you for your time and for sharing your thoughts and experiences with me. It will help us to understand better the status of COVID-19 vaccine access and uptake in refugee and migrant populations.
