# Supplementary material for: Deployment and uptake of COVID-19 vaccines for refugees and migrants in regular and irregular situations: a mixed-method multicountry study
Source: BMJ Open. 2025 Feb 2;15(1):e087629. doi: 10.1136/bmjopen-2024-087629 (PMC11904359; doi:10.1136/bmjopen-2024-087629)
Supplement: Supplementary file 4 [file bmjopen-2024-087629.reviewer_comments.pdf]

PEER REVIEW HISTORY

BMJ Open publishes all reviews undertaken for accepted manuscripts. Reviewers are asked to complete a checklist review form and are provided with free text boxes to elaborate on their assessment. These free text comments are reproduced below.

ARTICLE DETAILS

Title (Provisional)

Deployment and Uptake of COVID-19 Vaccines for Refugees and Migrants in Regular and Irregular Situations: a Mixed-Method Multi-Country Study

Authors

Benavente, Pierina; Kampalath, Vinay N.; Zan, Moussa Lonkila; Tran, Nguyen Toan; Czapka, Elżbieta Anna; Hosseinalipour, Seyed-Moeen; Teran, Enrique; Martens, Cheryl; Karmacharya, Biraj Man; Joshi, Anjali; Das, Jai K.; Padhani, Zahra A.; Jurlano, Vicente B.; Kabamalan, Maria Midea M.; Nyirazinyoye, Laetitia; Blanchet, Karl

---

VERSION 1 - REVIEW

**Reviewer**	**1**
**Name**	**M. Thomas, Christine**
**Affiliation**	**Tennessee Department of Health**
**Date**	**26-Jun-2024**
**COI**	**None to disclose.**

The authors conducted a thoughtful and detailed mixed-methods study assessing COVID-19 vaccine practices among migrants and refugees across five countries. The rigorous mixed methods facilitates both quantitative assessment of vaccine uptake and assessment of certain barriers and facilitators while also accounting for themes identified deductively and inductively through qualitative analysis of interviews and focus group discussions with key informants in each country. Their detailed findings showcase the nuance and heterogeneity of these communities geographically and with regards to demographic and social factors. In my opinion, this is a well-done study and manuscript and provides helpful information not only for academia but for ongoing and future efforts to equitably provide vaccines.

Regarding the qualitative analysis of policies (page 13, lines 56-58). Did the authors do any content analysis as part of this? I'm curious if there were differences on emphasis of inclusivity (such as was equity in distribution referenced more frequently or in more documents for certain countries). I'm also curious if there were any differences in language

used in the policies (for example were policies stating equity is important or outlining how to implement strategies for equity such as ensuring language access).

Did the authors account at all for limitations with a chronologically phased study approach during a rapidly evolving public health response. I think this limitation would be minimal given the quick timeframe in which both phases were conducted, but was there any indication that vaccines were more widely available during the months prior to survey administration compared to months prior to KII and FGD in a manner that could cause differential findings between the quantitative and qualitative analyses?

A few minor points for manuscript clarity:

- Abstract: Could the authors more clearly identify which results belong to the qualitative phase versus survey results? For example, do lines 31-36 came from qualitative phase (I presume it did) versus if these were survey questions?

- Methods: could the authors define the variable for “formal education not completed” (results page 10, line 6). For example, does this include only persons without any primary education?

- Figure 2: Typo in the title word “Health”

- Table 4: Column heading for Pakistan missing “n=”

**Reviewer**	**2**
**Name**	**El Arab, Rabie Adel**
**Affiliation**	**Almoosa College of Health Sciences**
**Date**	**07-Aug-2024**
**COI**	**None**

Dear Authors,

Thank you for the opportunity to review your manuscript titled "Deployment and Uptake of COVID-19 Vaccines for Refugees and Migrants in Regular and Irregular Situations: a Mixed-Method Multi-Country Study." Your research provides valuable insights into the challenges and successes of vaccinating vulnerable populations during the COVID-19 pandemic. I have outlined several comments and suggestions below that I believe will help strengthen your manuscript.

Data Interpretation and Estimation

Self-Reported Vaccination Status: The reliance on self-reported data can lead to overestimation. It would be beneficial to discuss this limitation in more detail in the manuscript and, where possible, mention any efforts made to verify self-reported data against official records.

Calculation of COVID-19 Vaccine Equity Index (CVEI): While innovative, please ensure the assumptions and methodology for CVEI calculation are clearly justified and transparent. Clarify the potential limitations and the rationale behind the CVEI calculation in the discussion section.

Sampling Bias: The use of snowball sampling for Migrants in Irregular Situations (MIIS) might result in a non-representative sample. Acknowledge this limitation more explicitly in the limitations section.

Non-Stratified Analysis: The lack of stratification by key characteristics (e.g., gender, migration status) may obscure important variations within the sample. While further stratification might not be possible post hoc, discussing this limitation and its potential impact on findings would be beneficial.

Introduction

Incorporate more recent literature to strengthen the context regarding vaccination efforts and challenges faced by refugees and migrants during the COVID-19 pandemic.

Methodology

While the qualitative sampling strategy is well-justified, the quantitative sampling could benefit from a more systematic approach to reduce bias. More details on the validation of the codebook for hybrid coding would enhance transparency.

Results

Present the findings on barriers and facilitators with more emphasis on the differences between countries to add depth. Provide a clearer explanation of the CVEI and its calculation for readers unfamiliar with the concept.

Discussion

Include a more critical analysis of the limitations and their impact on the study's conclusions. Discuss the practical challenges of implementing the policy recommendations provided.

Conclusion

While succinct, reinforce the key findings and their implications for future research and policy. Address the limitations of the study more explicitly to provide a balanced view.

References

Ensure a balanced representation of sources from different regions to further strengthen the manuscript.

Confidence Intervals and P-values

Issue: Ensure that confidence intervals and p-values are reported for all key statistical comparisons to provide a measure of the precision and significance of your estimates.

Specific Issues:

Missing Confidence Intervals: For vaccination rates, barrier prevalence, and other key metrics, confidence intervals are either missing or inconsistently reported.

P-values: Not all p-values are reported for statistical comparisons, making it difficult to assess the significance of findings.

Recommendation:

Include Confidence Intervals: Report 95% confidence intervals for all key estimates (e.g., vaccination rates, percentages of participants facing barriers). This will provide a measure of precision and help readers understand the range within which the true population parameter lies.

Example: Instead of reporting "86.6% of respondents were vaccinated," report "86.6% (95% CI: 83.5% - 89.7%) of respondents were vaccinated."

Report P-values: Provide p-values for all hypothesis tests conducted. This includes comparisons between groups (e.g., vaccination rates between regular and irregular migrants, men vs. women).

Example: "The vaccination rate was significantly higher in refugees (93.6%) compared to regular migrants (81.3%), $p < 0.001$."

Handling Missing Data

Issue: The manuscript does not clearly state how missing data were handled, which can impact the robustness of the results.

Specific Issues:

Unclear Handling of Missing Data: The methodology section lacks a description of how missing data were managed, which can lead to biases if not properly addressed.

Impact of Missing Data: The potential impact of missing data on the study's findings is not discussed. Descriptive Statistics Misinterpreted as Inferential: Some conclusions are drawn from descriptive statistics without appropriate inferential tests.

Lack of Hypothesis Testing: There is a need for clear differentiation between description (e.g., prevalence rates) and inference (e.g., comparing groups).

Recommendation:

Differentiate Clearly: Clearly distinguish between descriptive and inferential statistics in your results section.

Example: "Descriptive statistics indicated that 30.8% of respondents were concerned about vaccine reactions. Inferential statistics showed a significant difference in this concern between men and women ($p = 0.02$)."

Use Appropriate Tests: Ensure that conclusions about group differences are supported by appropriate statistical tests (e.g., chi-square tests, t-tests, ANOVA).

Example: "A chi-square test indicated that the difference in vaccination rates between regular migrants (81.3%) and irregular migrants (88.6%) was statistically significant (χ^2 = 6.72, p = 0.01)."

Multivariable Analysis

Issue: The manuscript primarily relies on univariable analyses, which may not account for potential confounding variables.

Specific Issues:

Potential Confounding: Key relationships may be confounded by other variables (e.g., age, education, duration in host country).

Lack of Adjusted Estimates: Without multivariable analysis, it's unclear whether observed associations hold after adjusting for confounders.

Recommendation:

Perform Multivariable Analyses: Use multivariable regression models (e.g., logistic regression, linear regression) to adjust for potential confounders.

Example: "A logistic regression model was used to assess the association between migration status and vaccination uptake, adjusting for age, gender, education level, and duration in the host country."

Report Adjusted Results: Present both unadjusted and adjusted estimates to show the impact of confounding variables.

Example: "After adjusting for confounders, the association between irregular migration status and higher vaccination uptake remained significant (aOR = 1.45, 95% CI: 1.10 - 1.92, p = 0.01)."

I commend you on the important work you have done and look forward to seeing the revised version of your manuscript.

Best regards

VERSION 1 - AUTHOR RESPONSE

REVIEWER 1

The authors conducted a thoughtful and detailed mixed-methods study assessing COVID-19 vaccine practices among migrants and refugees across five countries. The rigorous mixed methods facilitates both quantitative assessment of vaccine uptake and assessment of certain barriers and facilitators while also accounting for themes identified deductively and inductively through qualitative analysis of interviews and focus group discussions with key informants in each country. Their detailed findings showcase the nuance and heterogeneity of these communities geographically and with regards to demographic and social factors. In my opinion, this is a well-done study and manuscript and provides helpful information not only for academia but for ongoing and future efforts to equitably provide vaccines.

Comment 1: Regarding the qualitative analysis of policies (page 13, lines 56-58). Did the authors do any content analysis as part of this? I'm curious if there were differences on emphasis of inclusivity (such as was equity in distribution referenced more frequently or in more documents for certain countries). I'm also curious if there were any differences in language used in the policies (for example were policies stating equity is important or outlining how to implement strategies for equity such as ensuring language access).

Response: Thank you for this valuable feedback. Regarding the analysis of policies, we reviewed specific aspects of the policies and compared them among countries. We have added these details in the manuscript (lines 21 to 23 in page 6):
"Instead of conducting a formal content analysis, specific aspects of the policies were reviewed and compared among countries."

Concerning the emphasis of inclusivity, we have added more details about different countries in the Political commitment section (under Results, page 15):
Lines 4 to7 in page 15*: "For instance, the Ecuadorian vaccination program 9/1000, launched at the beginning of the Guillermo Lasso government on May 24, 2021, aimed to vaccinate 9 million people within the first 1000 days of his term and was the first in the region to make vaccines available to its migrant population."*
Lines 39 to 40 in page 15: *"...such as the use of election cards in Ecuador and the national database through National Database and Registration Authority in Pakistan"*
Lines 42 to 44 in page 15: *"Community representatives in Pakistan reported instances where COVID-19 vaccination cards were sold to some migrant groups who did not want to be vaccinated to avoid registration by national authorities."*

Regarding the language aspect, we did not analyse this specific point in the policy analysis. However, during the qualitative interviews the KIs they reported on the translation of information and how different organizations played a role in this process. We have added this paragraph in the Result section under Facilitators/Adequate sources and provision of information (line 52 in page 13 to line 2 in page 14):
"Regarding the language, KIs from Pakistan, Nepal, Rwanda, and the Philippines indicated that local and international government organizations, in coordination with local governments, facilitated the translation of information into different languages to reach the specific migrant and refugee groups in their respective countries. In Ecuador, since the majority of migrants and refugees speak the same language, this was not a significant issue."

Comment 2: Did the authors account at all for limitations with a chronologically phased study approach during a rapidly evolving public health response. I think this limitation would be minimal given the quick timeframe in which both phases were conducted, but was there any indication that vaccines were more widely available during the months prior to survey administration compared to months prior to KII and FGD in a manner that could cause differential findings between the quantitative and qualitative analyses?

Response: Thank you for your valuable feedback. We agree that the chronologically phased study approach is a limitation of our study that could have caused differential finding between the quantitative and qualitative analysis. We have added this a limitation in the discussion section (lines 6 to 11in page 18):
"Additionally, our study was conducted in different phases during a rapidly changing health crisis. Although the short timeframe between the phase I and II of our study helped mitigate some of the issues, differences in vaccine availability between the KIs interviews and FGDs and the survey period could potentially have impacted our findings. This may have led to

variations between our quantitative and qualitative analyses, with the qualitative feedback generally being more critical of the vaccine rollout compared to the quantitative results."
**Comment 3:** Abstract: Could the authors more clearly identify which results belong to the qualitative phase versus survey results? For example, do lines 31-36 came from qualitative phase (I presume it did) versus if these were survey questions?<br><br>**Response:** Thank you for your comment. We made some changes in lines 20 to 27 of page 2 to clearly identified which results belong to the qualitative phase versus the quantitative one: New version: *"However, **the qualitative phase reported** delays and inequities in the early stage of the vaccination process in all countries. Overall, 64.2% of respondents perceived that government' campaigns were successful. Both the qualitative and quantitative phases identified several barriers and facilitators. The main barriers included a lack of trust in authorities, extended waiting times and distance to vaccination centres, discrimination and xenophobia, lack of identity documentation, and adverse reaction concerns. **On the other hand, the primary facilitators were** the widespread distribution of vaccination centres, adequate sources and provision of information, specific campaigns for refugees and migrants, free vaccination, and the motivation to protect others' health."*
**Comment 4:** Methods: could the authors define the variable for "formal education not completed" (results page 10, line 6). For example, does this include only persons without any primary education?<br><br>**Response:** Thank you for your comment. We have added this clarification in the result section (line 34 in page 8)
**Comment 5:** Figure 2: Typo in the title word "Health"<br><br>**Response:** Thank you for pointing this out. We corrected this typo in figure 2 of the supplementary material.
**Comment 6**: Table 4: Column heading for Pakistan missing "n="<br><br>**Response:** Thank you for pointing this out. We added this information in Table 4 of the supplementary material.

REVIEWER 2

Dear Authors,

Thank you for the opportunity to review your manuscript titled "Deployment and Uptake of COVID-19 Vaccines for Refugees and Migrants in Regular and Irregular Situations: a Mixed-Method Multi-Country Study." Your research provides valuable insights into the challenges and successes of vaccinating vulnerable populations during the COVID-19 pandemic. I have

outlined several comments and suggestions below that I believe will help strengthen your manuscript.

**Comments 1 to 4: Data Interpretation and Estimation**
**Comment 1:** Self-Reported Vaccination Status: The reliance on self-reported data can lead to overestimation. It would be beneficial to discuss this limitation in more detail in the manuscript and, where possible, mention any efforts made to verify self-reported data against official records.<br><br>**Response:** Thank you for this valuable feedback. We agree that self-reported vaccination status is a limitation of our study, and we have added more details about this in the discussion section (lines 50 to 53 in page 17):<br>*"Thirdly, the reliance on self-reported information, including vaccination status, poses a risk of recall bias or information concealment, which can lead to overestimation or inaccuracies in the reported data. Due to the nature and design of the study, we were unable to verify the self-reported data against official verification records."*
**Comment 2:** Calculation of COVID-19 Vaccine Equity Index (CVEI): While innovative, please ensure the assumptions and methodology for CVEI calculation are clearly justified and transparent. Clarify the potential limitations and the rationale behind the CVEI calculation in the discussion section.<br><br>**Response:** Thank you for this comment. We agreed that we need to clarify the potential limitation and the rationale behind the CVEI calculation in the discussion section. We added this paragraph under limitations and strengths (lines 13 to 18 in page 18)<br>*"Furthermore, the calculation of the COVID-19 Vaccine Equity Index (CVEI) was based on comparing the proportion of unvaccinated individuals in subgroups to the total population. This method assumes reliable data collection and representativeness of the sampled populations. However, variations in data reporting and potential sampling bias may affect the accuracy of the CVEI. Despite these challenges, the CVEI provides a valuable measure of vaccination equity, highlighting disparities that need to be addressed."*
**Comment 3:** Sampling Bias: The use of snowball sampling for Migrants in Irregular Situations (MIIS) might result in a non-representative sample. Acknowledge this limitation more explicitly in the limitations section.<br><br>**Response**: Thank you for this valuable feedback. We have added more details to be more explicit about this limitation in the discussion section (lines 36 to 40 in page 17):<br>*"Specifically, reaching MIIS required a distinct approach, necessitating a snowball sampling method. This technique can result in a non-representative sample, introducing the possibility that our MIIS sample may not accurately reflect the broader MIIS population. This limitation should be considered when interpreting the findings, as it may impact the generalizability of our results."*
**Comment 4:** Non-Stratified Analysis: The lack of stratification by key characteristics (e.g., gender, migration status) may obscure important variations within the sample. While further stratification might not be possible post hoc, discussing this limitation and its potential impact on findings would be beneficial.

Response: Thank you so much for your comments. We have added more details about the non-stratified analysis in discussion section (lines 44 to 48 in page 17):
"Secondly, the main target group was not stratified by key characteristics, such as gender and migration status. The absence of stratified analysis could mask important variations within the sample, potentially leading to an incomplete understanding of the differences between various subgroups. Consequently, MIRS in Rwanda were inadequately represented in the sample."

Comment 5: Introduction: Incorporate more recent literature to strengthen the context regarding vaccination efforts and challenges faced by refugees and migrants during the COVID-19 pandemic.

Response: Thank you for your comment. We have added a new paragraph to the background section to specifically address this comment. While most findings regarding vaccine barriers and acceptance among refugee and migrant communities and proposals to remove barriers remain the same as previous COVID-19 vaccination studies, we cited 5 newer publications (references 8 to 11) here and specifically mentioned some emergent findings, including 1 study from late 2023 and 3 studies from 2024 (lines 18 to 32 in page 3):
"Recent studies highlight the importance of tailored vaccination models to meet the needs of diverse populations, emphasizing the need for flexible funding, outreach, translation, and culturally responsive services to address ongoing and emergent factors such as vaccine hesitancy. Considering the slower and lower levels of uptake among migrants in irregular situations, even in the few European countries who provided equal access to COVID-19 vaccines, shows that sole focus on policy commitments, cannot guarantee uptake and adoptive vaccine programs for different sub-communities are needed. Some recent findings indicate delayed, overdue or missed follow-up COVID-19 shots among non-European migrants and resettled refugees in England, especially among older and non-white groups, indicating a need for further research. Moreover, lower perception of infection and lower integration level into host country are also among reasons for lower vaccine acceptance. Additionally, the World Health Organization (WHO) has published interim guidance on COVID-19 immunization in refugees and migrants, outlining key principles and policy considerations for equitable prioritization and access to vaccines. These efforts underscore the importance of community engagement, communication to build trust, and innovative approaches for vaccine delivery."

Comment 6: Methodology: While the qualitative sampling strategy is well-justified, the quantitative sampling could benefit from a more systematic approach to reduce bias. More details on the validation of the codebook for hybrid coding would enhance transparency.

Response: Thank you for pointing this out. We have added more details regarding the quantitative sampling in page 7 (lines 15 to 19):
"To enhance the rigor of our quantitative sampling and reduce bias, we implemented a range of strategies tailored to the specific circumstances and challenges present in each country. For example, in some countries, convenient sampling was employed due to accessibility and proximity, while in others, initial seeds provided by humanitarian organizations facilitated snowball sampling. Additionally, purposive sampling was used in cities with high concentrations of migrant populations."

Regarding the details on the validation of the codebook for hybrid coding, we have added more details to enhance transparency (lines 13 and 15 in page 6):

"Consensus coding was used to enhance the trustworthiness of the analysis. The researchers from the national teams and the core research group coded the same transcripts and compared results."
**Comment 7: Results:** Present the findings on barriers and facilitators with more emphasis on the differences between countries to add depth. Provide a clearer explanation of the CVEI and its calculation for readers unfamiliar with the concept.<br><br>**Response:** Thank you for your valuable feedback. We agree that highlighting more differences among the countries would add depth to our manuscript. We have added more information comparing countries throughout the results section.<br>In addition, we have provided more details about the CVEI on page 9 (lines 10 to 11) to make it easier for readers to understand: *"Overall, the CVEI was 0.824, suggesting that vaccine coverage was generally higher among migrants compared to the general population globally."*
**Comment 8: Discussion:** Include a more critical analysis of the limitations and their impact on the study's conclusions. Discuss the practical challenges of implementing the policy recommendations provided.<br><br>**Response:** Thank you for your comment. We have included more details in the discussion section on the limitations of our study and its impact on the study's conclusions according to your feedback (please see responses to comment 2 from reviewer 1, and comments 1, 3, 4, 14 from reviewer 2)<br><br>In addition, we have added more information about the challenges of implementing policy recommendations provided (line 23 to 31 in page 17):<br>*"While our study provides valuable insights to inform policy recommendations and enhance vaccine uptake among refugees and migrants, several practical challenges must be acknowledged. Simplifying the vaccination registration process can be hindered by bureaucratic procedures. Designing and implementing targeted campaigns requires addressing diverse cultural and linguistic needs. Deploying mobile vaccination teams presents logistical challenges such as funding and vaccine storage. Building trust among refugees and migrants who may have experienced past traumas or distrust towards authorities is a complex process. Combatting misinformation and vaccine hesitancy demands continuous effort and reliable information sources. By recognizing these challenges, policymakers can better implement recommendations to ensure equitable vaccine access and health service delivery."*
**Comment 9: Conclusion:** While succinct, reinforce the key findings and their implications for future research and policy. Address the limitations of the study more explicitly to provide a balanced view.<br><br>**Response:** Thank you for your comment. We have reinforced the key findings and their implications for future research and have addressed the limitations in our conclusions to provide a balance view (lines 35 to 45 in page 18):<br>*"Future research should focus on identifying and addressing barriers such as long wait times, distance to vaccination sites, and trust issues. Targeted strategies like simplified registration processes, targeted campaigns, and mobile vaccination teams, can enhance vaccine uptake. Building trust with host country authorities and healthcare providers is also crucial to improve vaccine acceptance among refugees and migrants.*

Despite limitations such as diverse recruitment methods and reliance on self-reported data, our study provides valuable insights into vaccine coverage among refugees and migrants. Addressing these challenges is essential for future vaccine deployments and health service delivery, helping to build more resilient and inclusive health systems for populations in vulnerable situations, particularly during health crises like the pandemic."

Comment 10: References: Ensure a balanced representation of sources from different regions to further strengthen the manuscript.

Response: Thank you for your comment. We feel this has been fairly addressed automatically by incorporating more recent studies in our literature review in response to your comment 5, which also includes a systematic review concluded in 2024 that incorporated studies from different regions. We had also tried to include evidence from different regions when collecting existing evidence from literature and did not exclude based on region. Considering the target countries of the research, we also feel a good representation has been achieved, especially having local teams leading the country research profiles.

Comment 11 to 12: Confidence Intervals and P-values

Ensure that confidence intervals and p-values are reported for all key statistical comparisons to provide a measure of the precision and significance of your estimates.

Specific Issues:

Comment 11: Missing Confidence Intervals: For vaccination rates, barrier prevalence, and other key metrics, confidence intervals are either missing or inconsistently reported.
Recommendation: Include Confidence Intervals: Report 95% confidence intervals for all key estimates (e.g., vaccination rates, percentages of participants facing barriers). This will provide a measure of precision and help readers understand the range within which the true population parameter lies.
Example: Instead of reporting "86.6% of respondents were vaccinated," report "86.6% (95% CI: 83.5% - 89.7%) of respondents were vaccinated."

Response: Thank you for your valuable feedback. Based on your suggestion, we have now included confidence intervals (CIs) in our result section. Adding these CIs allows us to present a more comprehensive picture of our findings, as confidence intervals indicate the range within which we expect the true effect size to lie with a specified level of confidence (typically 95%). Thank you again for highlighting this point, which has strengthened the clarity and interpretability of our analysis.

Comment 12: P-values: Not all p-values are reported for statistical comparisons, making it difficult to assess the significance of findings.
Recommendation: Report P-values: Provide p-values for all hypothesis tests conducted. This includes comparisons between groups (e.g., vaccination rates between regular and irregular migrants, men vs. women).
Example: "The vaccination rate was significantly higher in refugees (93.6%) compared to regular migrants (81.3%), $p < 0.001$."

Response: Thanks for this remark. We added Chi2 Significance p-values for crosstabulations in the result section.

**Comment 13 to 15: Handling Missing Data**<br>The manuscript does not clearly state how missing data were handled, which can impact the robustness of the results.<br>Response: Thanks for pointing this out.
Specific Issues:<br>**Comment 13:** Unclear Handling of Missing Data: The methodology section lacks a description of how missing data were managed, which can lead to biases if not properly addressed.<br><br>**Response:** Thank you for pointing this out. In response to your feedback, we have added sentences in the Methods section to clarify our approach to handling missing data. Specifically, we have provided details on the techniques and strategies we used to manage missing values, ensuring transparency in how data gaps were addressed.
**Comment 14:** Impact of Missing Data: The potential impact of missing data on the study's findings is not discussed. Descriptive Statistics Misinterpreted as Inferential: Some conclusions are drawn from descriptive statistics without appropriate inferential tests.<br><br>**Response**: The primary objective of our study was to conduct a descriptive analysis, focusing on summarizing key characteristics and patterns within the data to provide a clear overview of the variables under investigation. Our initial goal was to outline these trends and insights without extending the analysis to broader inferences. However, it's important to clarify that we cannot reliably generalize our results to any specific population or draw inferences at a national level. We have added the following comment in the discussion section (lines 40 to 43 in page 17): "*The nature of the target population, comprising regular and irregular migrants, as well as refugees, did not permit the use of a sampling frame for selecting respondents. This limitation affects the representativeness of the sample and prevents us from conducting inferential analysis at any level.*"
**Comment 15:** Lack of Hypothesis Testing: There is a need for clear differentiation between description (e.g., prevalence rates) and inference (e.g., comparing groups).<br><br>**Response:** Thank you for this valuable observation. We appreciate your thoughtful feedback and agree on the importance of addressing the points you raised. In light of your suggestions, we are committed to carefully considering your recommendations to improve the rigor and clarity of our study.<br><br>Throughout the manuscript, we have added p-values from the Chi-square (Chi^2) test to accompany our cross-tabulations, providing statistical support for the relationships described in our analysis. This inclusion allows us to demonstrate whether the observed associations between categorical variables are statistically significant, giving readers a clearer understanding of the strength and relevance of these relationships. We chose the Chi-square test over the t-test because the latter is limited to comparing means between two groups or categories. Given that our analysis includes variables with more than two categories, the Chi-square test is a more suitable option. The Chi-square test allows us to evaluate relationships within a broader range of categorical groups, enabling a comprehensive examination of the associations in our data.
**Comment 16 to 17: Multivariable Analysis**<br>The manuscript primarily relies on univariable analyses, which may not account for potential confounding variables.

Specific Issues:
Comment 16: Potential Confounding: Key relationships may be confounded by other variables (e.g., age, education, duration in host country).
Recommendation: Perform Multivariable Analyses: Use multivariable regression models (e.g., logistic regression, linear regression) to adjust for potential confounders.
Example: "A logistic regression model was used to assess the association between migration status and vaccination uptake, adjusting for age, gender, education level, and duration in the host country."

Response: Thank you for bringing this to our attention. While we acknowledge that performing regression analysis with covariates can be highly valuable in adjusting for potential confounding factors and isolating the effects of specific variables, the primary intent of this paper is to remain descriptive. Our focus in this study is to provide a clear, straightforward overview of the observed data without extending into causal inference or complex adjustments. By emphasizing a purely descriptive approach, we aim to highlight general patterns, distributions, and associations within the dataset, providing readers with an accessible, high-level understanding of the variables of interest.

Comment 17: Lack of Adjusted Estimates: Without multivariable analysis, it's unclear whether observed associations hold after adjusting for confounders.
Recommendation: Report Adjusted Results: Present both unadjusted and adjusted estimates to show the impact of confounding variables.
Example: "After adjusting for confounders, the association between irregular migration status and higher vaccination uptake remained significant (aOR = 1.45, 95% CI: 1.10 - 1.92, p = 0.01)."
I commend you on the important work you have done and look forward to seeing the revised version of your manuscript.

Response: Thanks for pointing this out. Adding regression with covariates would indeed offer more detailed insights into potential confounding effects, but this would shift the study's focus from description to interpretation and inference, which is beyond our intended scope. For this paper, we have opted to keep the analysis straightforward and descriptive, allowing for an unbiased presentation of the data as it stands. Thank you again for this insightful suggestion. We appreciate your understanding of our approach and focus in this work.

We have attached the updated manuscript and supplementary material, including the clean versions and the one with tracked changes to facilitate the revision.

Pierina Benavente, on behalf of the authors
